# Targeting HDAC and PARP Enhances STING‐Dependent Antitumor Immunity in STING‐Deficient Tumor

**DOI:** 10.1002/advs.202507904

**Published:** 2025-08-11

**Authors:** Chengzhou Mao, Weiwen Fan, Jiaqi Liu, Fangzhou Yang, Wenkai Li, Lulu Li, Zhichao Shi, Qinyuan Li, Zigao Yuan, Yuyang Jiang, Bizhu Chu

**Affiliations:** ^1^ Department of Anatomy and Histology Shenzhen University Medical School Shenzhen University Shenzhen 518055 China; ^2^ Guangdong Provincial Key Laboratory of Chinese Medicine Ingredients and Gut Microbiomics School of Pharmacy Shenzhen University Medical School Shenzhen University Shenzhen 518055 China; ^3^ Present address: Department of Pharmacy The Second Hospital of Longyan Longyan 364099 China; ^4^ Institute of Biomedical Health Technology and Engineering Shenzhen Bay Laboratory Shenzhen 518132 China; ^5^ State Key Laboratory of Chemical Oncogenomics Tsinghua Shenzhen International Graduate School Shenzhen 518055 China

**Keywords:** cGAS–STING pathway, HDAC, Immunotherapy, PARP, tumor immune microenvironment

## Abstract

The stimulator of interferon genes (STING)‐mediated innate immune pathway plays an important role in tumor immunosurveillance. STING deficiency in tumors impairs the interferon response; however, the underlying mechanism remains unclear. Here, it is demonstrated that histone deacetylase (HDAC) suppresses STING expression by reducing H3K9 acetylation at the STING promoter. The combined inhibition of HDAC and poly(ADP‐ribose) polymerase (PARP) induced STING re‐expression and promoted cytosolic DNA accumulation, which further activated the interferon response in STING‐deficient tumors. A bifunctional HDAC and PARP inhibitor displayed potent antitumor immunity by reinducing and activating the STING pathway. Mechanistically, the bifunctional HDAC and PARP inhibitor induced “BRCAness,” thereby restoring synthetic lethality, reactivating STING expression, and promoting the infiltration and activation of T cells and dendritic cells in the tumor microenvironment. Notably, STING depletion reversed the antitumor effect. Moreover, dual inhibition of HDAC and PARP significantly enhanced the antitumor immune response to immune checkpoint blockade by inducing adaptive immune memory. These findings underscore dual HDAC and PARP inhibition as a promising therapeutic strategy for overcoming the STING pathway deficiency and augmenting antitumor immunity in cancer.

## Graphical abstract

Dual HDAC and PARP inhibition restores STING signaling, enhances interferon response, and boosts antitumor immunity in STING‐deficient tumors.

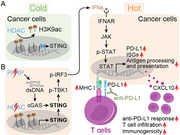

## 1 Introduction

Immune checkpoint blockade (ICB) treatment has demonstrated durable curative effects for multiple malignancies.^[^ ^1^, ^2^ ^]^ However, inadequate activation of the immune system significantly restricts cancer immunity, leading to suboptimal efficacy and low clinical response rates in patients with solid tumors.^[^ ^3^, ^4^ ^]^ The innate immune system not only serves as the first line of defense against pathogens but also plays a crucial role in initiating and sustaining T cell responses necessary for the efficacy of ICB therapy.^[^ ^5^ ^]^ The cyclic GMP–AMP synthase (cGAS)–stimulator of interferon genes (STING) signaling pathway is a critical component of innate immunity, making it an attractive target for antitumor immune responses.^[^ ^6^ ^]^ This pathway is activated by the presence of cytosolic DNA, leading to the induction of type I or III interferons (IFNs) through IFN regulatory factor 3 (IRF3), followed by Janus kinase (JAK)–signal transducer and activator of transcription (STAT)‐dependent expression of cytokines and a broad array of IFN‐stimulated genes (ISGs).^[^ ^7^, ^8^ ^]^ Ultimately, the secretion of IFNs and ISGs by tumor cells enhances antigen presentation and facilitates the recruitment and activation of cytotoxic T cells, thereby promoting the conversion of immunologically “cold” tumors into T cell‐enriched “‘hot”’ tumors capable of supporting effective antitumor immune responses.^[^ ^7^, ^9^, ^10^ ^]^

Targeting the DNA damage response (DDR) pathway enhances STING signaling‐mediated antitumor immune response.^[^ ^11^, ^12^, ^13^, ^14^, ^15^ ^]^ Several DDR inhibitors [e.g., poly(ADP‐ribose) polymerase (PARP) inhibitors] have been approved for treating multiple cancers.^[^ ^11^, ^16^, ^17^ ^]^ PARP inhibitors (PARPis) promote the accumulation of cytosolic DNA fragments, which activate the intracellular cGAS–STING pathway, thereby initiating innate immune responses and subsequently facilitating the activation of adaptive immunity.^[^ ^12^, ^13^, ^15^ ^]^ Despite their remarkable therapeutic efficacy, PARPi treatments are often limited by the emergence of *de novo* and acquired drug resistance.^[^ ^18^, ^19^ ^]^ Moreover, tumors often employ various immunosuppressive mechanisms for neutralizing the activation of innate immunity intrinsic to cancer.^[^ ^20^, ^21^ ^]^ For example, IFN signaling triggered by impaired DNA repair, chemoradiotherapy‐induced DNA damage, or chromatin dysregulation can lead to upregulation of the immune checkpoint regulator programmed death‐ligand 1 (PD‐L1), enabling cancer cells to evade immune surveillance.^[^ ^12^, ^13^, ^14^, ^22^ ^]^ Notably, cGAS or STING expression is remarkably low in tumor cells that often exhibit weak antitumor immune responses,^[^ ^23^, ^24^, ^25^ ^]^ highlighting the necessity for identifying alternative clinically relevant mechanisms to potentiate the efficacy of monotherapies or combination PARPi therapies.

STING is frequently downregulated in various cancers,^[^ ^26^, ^27^, ^28^ ^]^ implying that its silencing in tumor cells contributes to immune evasion and may limit the effectiveness of PARPi‐based therapies. In many cases, this downregulation occurs through epigenetic reprogramming rather than through loss‐of‐function mutations or deletion events.^[^ ^23^, ^28^ ^]^ Distinct epigenetic mechanisms are responsible for STING silencing in different tumor types. Inhibitors targeting DNA methyltransferase (DNMT) in gliomas and melanoma.^[^ ^23^, ^28^ ^]^ enhancer of Zeste homolog 2 (EZH2) in lung cancers,^[^ ^26^ ^]^ and lysine demethylase 5 (KDM5) in colorectal cancers^[^ ^24^ ^]^ are known to restore STING expression. Histone deacetylases (HDACs) are enzymes that modulate histone function by removing acetyl groups from lysine residues, thereby playing crucial roles in regulating gene transcription and contributing to aberrant gene expression in various cancers.^[^ ^29^ ^]^ HDAC inhibitors (HDACis) have become attractive targets for treating hematological or solid tumors in different phases of clinical trials.^[^ ^30^ ^]^ Although several HDACis have been approved by the US Food and Drug Administration (FDA) for various cancer types, their efficacy as monotherapies has been modest. Recent studies have investigated the potential benefits of combining HDACis with immunotherapy.^[^ ^29^, ^31^ ^]^ Although these reports suggest that HDACis can reprogram the tumor immune microenvironment, the underlying mechanism remains unclear. A synergistic effect of HDACi and PARPi has been observed in certain cancer types.^[^ ^32^, ^33^ ^]^ Previous studies have focused on the ability of combined treatments for promoting neoantigen generation, activating the cGAS–STING pathway, and/or restoring synthetic lethality. However, the mechanism by which these agents trigger an antitumor immune response, particularly by modulating the STING‐mediated innate immune response, is not fully understood.

In this study, we identified frequent loss of STING expression in pancreatic tumors and systematically screened epigenetic inhibitors for identifying potential regulators of intratumoral STING. Among the tested compounds, only HDACis robustly induced intratumoral STING expression by increasing acetylation at the STING promoter. Moreover, HDAC inhibition, in combination with PARP inhibition, elicited a robust tumor‐intrinsic innate immune response characterized by increased infiltration of T cells and dendritic cells (DCs) within the tumor microenvironment, ultimately enhancing the efficacy of ICB therapy. A bifunctional HDAC and PARP inhibitor (hereafter referred to as P2) upregulated STING expression in pancreatic cancer cells, accompanied by cytosolic DNA accumulation and activation of the tumor‐intrinsic cGAS–STING pathway, as evidenced by increased phosphorylation of TANK‐binding kinase 1 (TBK1) and elevated production of type I and III IFNs. Furthermore, P2 treatment elicited a robust antitumor immune response in a STING‐dependent manner, promoting T cell infiltration, DC activation, and improved immunotherapeutic efficacy of PD‐L1 blockade in syngeneic mouse models. Our findings highlight the potential of bifunctional HDAC and PARP inhibition to restore and activate cGAS–STING signaling and enhance responses to immunotherapy in STING‐deficient tumors.

## 2 Results

### 2.1 HDAC‐Mediated Silencing Contributes to STING Deficiency and Impaired Tumor‐Intrinsic Innate Immune Responses

**Figure 1:**
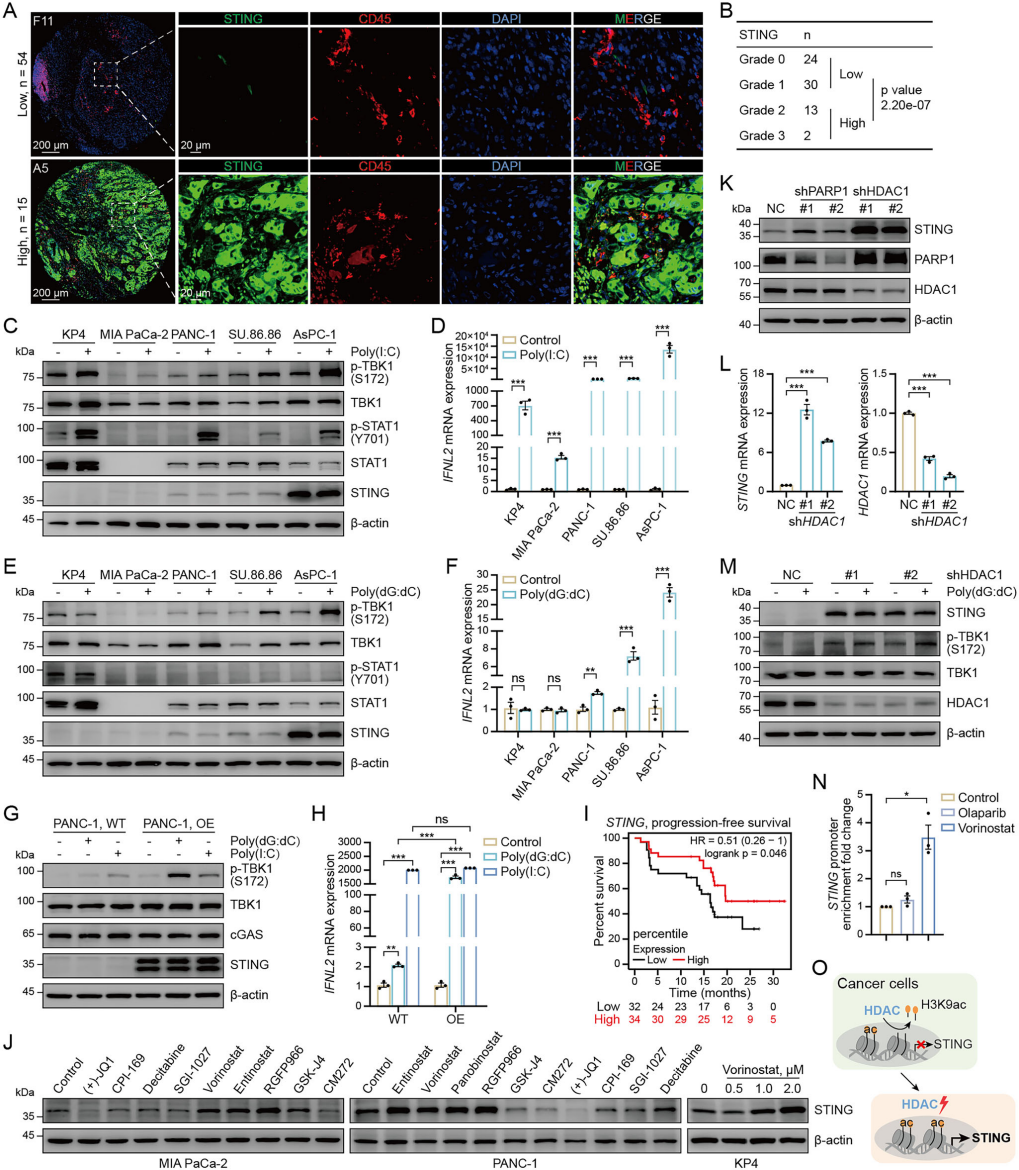
HDAC‐mediated silencing contributes to STING deficiency and impaired tumor‐intrinsic innate immune responses. A) Representative multiplex immunofluorescence images of pancreatic cancer tissue microarray showing STING (green), and CD45 (red) expression in tumor tissues. DAPI (blue) was used for nuclear counterstaining; *n* = 69. B) Quantification of pancreatic cancer tissue microarray samples stratified by STING expression scores; *n* = 69. C) Innate immune signaling‐related protein expression levels in pancreatic cancer cells using immunoblotting after transfection with 1 µg mL^−1^ Poly(I:C); n ≥ 3. D) Type III IFNs transcript levels were analyzed in pancreatic cancer cells using RT–qPCR after transfection with Poly(I:C); n ≥ 3. E) Innate immune signaling‐related proteins expression levels in pancreatic cancer cells transfection with 1 µg mL^−1^ Poly(dG:dC); n ≥ 3. F) Type III IFNs transcript levels in pancreatic cancer cells transfection with Poly(dG:dC); n ≥ 3. G) Immunoblot analysis of indicated proteins in PANC‐1 cells overexpressing STING and transfection with 1 µg mL^−1^ Poly(dG:dC) or Poly(I:C); n ≥ 3. H) RT–qPCR analysis of Type III IFNs transcripts in PANC‐1 cells following STING overexpression and transfection with Poly(dG:dC) or Poly(I:C); n ≥ 3. I) Kaplan–Meier progression‐free survival curves stratified by STING expression levels in patients undergoing immunotherapy; *n* = 66. J) The low‐expressing STING cell lines were treated with a series of epigenetic inhibitors for 48 h and were analyzed by immunoblotting; n ≥ 3. K–L) Immunoblot and RT–qPCR analyses of STING expression following HDAC1 and PARP1 knockdown in MIA PaCa‐2 cells; n ≥ 3. M) Knockdown of HDAC1 followed by Poly(dG:dC) transfection promoted TBK1 phosphorylation in MIA PaCa‐2 cells; n ≥ 3. N) ChIP analysis of MIA PaCa‐2 cells treated with Vorinostat (0.5 µm) or Olaparib (10.0 µm) for 48 h; n ≥ 3. O) Schematic of HDACis‐mediated epigenetic reactivation of STING expression in tumor cells. The data are expressed as mean ± standard error of the means (SEMs); unpaired student's *t*‐test; ns, not significant; ^*^ *p* < 0.05; ^**^ *p* < 0.01; ^***^ *p* < 0.001.

Activated tumor‐intrinsic innate immune signaling plays a vital role in antitumor immunity.^[^ ^11^ ^]^ This signaling pathway is triggered by pattern‐recognition receptors, such as retinoic acid‐inducible gene I (RIG‐I)/melanoma differentiation‐associated protein 5 (MDA5) and cGAS, which detect cytosolic RNA or DNA fragments, leading to the expression of IFNs and JAK–STAT‐dependent chemokines such as CCL5 and CXCL10 (Figure S1A, Supporting Information).^[^ ^7^, ^34^ ^]^ To assess whether innate immune signaling is defective in response to nucleoside analogs in tumor cells, we examined a panel of human pancreatic cancer cell lines and observed roughly normal cGAS expression, whereas STING was barely detectable in MIA PaCa‐2, KP4, PANC‐1, and SU.86.86 cells (Figure S1B, Supporting Information). Further analysis of gene expression profiles from The Human Protein Atlas (HPA) database corroborated these findings (Figure S1C,D, Supporting Information), and the majority of pancreatic cancer cell lines exhibited low STING expression (Figure S1E, Supporting Information). Next, we assessed STING expression using a pancreatic cancer tissue microarray comprising samples from 69 patients. Consistent with the findings in cancer cell lines, reduced or absent STING expression was observed in 54 of the 69 tumor specimens (**Figure** **1**A,B; Figure S1F, Supporting Information), suggesting that STING loss is a common feature in both pancreatic cancer tissues and cell lines.

To assess the relationship between STING expression and function, we transfected cells with the synthetic double‐stranded DNA (dsDNA) analog Poly(dG:dC) for activating the STING signaling pathway, or with the double‐stranded RNA (dsRNA) analog Poly(I:C) to trigger the RIG‐I signaling pathway. We evaluated the activation of the innate immune pathway effectors TBK1 and STAT1 in these STING‐deficient cells; AsPC‐1 cells were used as a positive control for ensuring that the nucleoside analogs were properly working. Treatment with Poly(I:C) significantly increased the phosphorylation of TBK1 and STAT1, and the mRNA levels of type I and III IFNs (Figure 1C,D; Figure S2A, Supporting Information). In contrast, activation of TBK1, STAT1, and IFNs was not observed when cells were treated with Poly(dG:dC) (Figure 1E,F; Figure S2B, Supporting Information). Lentivirus‐mediated STING overexpression in PANC‐1 cells enhanced TBK1 phosphorylation and IFN induction upon Poly(dG:dC) stimulation (Figure 1G,H). Furthermore, Kaplan–Meier analysis revealed that high STING expression was associated with improved prognosis across multiple cancer types in patients receiving immunotherapy (Figure 1I). Collectively, these results indicate that STING deficiency broadly impairs cytosolic DNA sensing and downstream innate immune signaling in human cancers.

To investigate whether STING expression is epigenetically silenced and can be restored by pharmacological intervention, we screened a panel of epigenetic inhibitors. Notably, only HDACis, including the pan‐HDACis Vorinostat and Panobinostat, the class I HDAC1 and HDAC3 inhibitor Entinostat, and the HDAC3 selective inhibitor RGFP966, significantly induced STING re‐expression (Figure 1J). Consistent with these findings, knockdown of class I and IIb *HDAC*s effectively restored STING expression, whereas silencing of class IIa or IV *HDAC*s showed no such effect (Figure 1K,L; Figure S2C–E). These observations were further verified by treatment with isoform‐selective HDACis, and the results demonstrated that class I and IIb HDACis significantly upregulated STING expression (Figure S2F, Supporting Information). To determine whether this effect was specific to pancreatic cancer, we treated other tumor cell lines with Entinostat. The STING‐upregulating capacity of Entinostat was not restricted to pancreatic cancer, and was also observed in breast cancer cells (Figure S2G, Supporting Information). Furthermore, activation of the STING pathway was confirmed by increased phosphorylated (phospho) TBK1 (S172) levels following Poly(dG:dC) stimulation in *HDAC1*‐silenced cells (Figure 1M). Chromatin immunoprecipitation (ChIP) assays revealed elevated H3K9 acetylation at the STING promoter in Vorinostat‐treated cells (Figure 1N), indicating enhanced transcriptional accessibility. Together, these results suggest that class I and IIb HDACs repress STING expression by deacetylating H3K9 at its promoter (Figure 1O).

### 2.2 HDAC and PARP Inhibitors Synergistically Activate STING Signaling in STING‐Defective Cells

**Figure 2:**
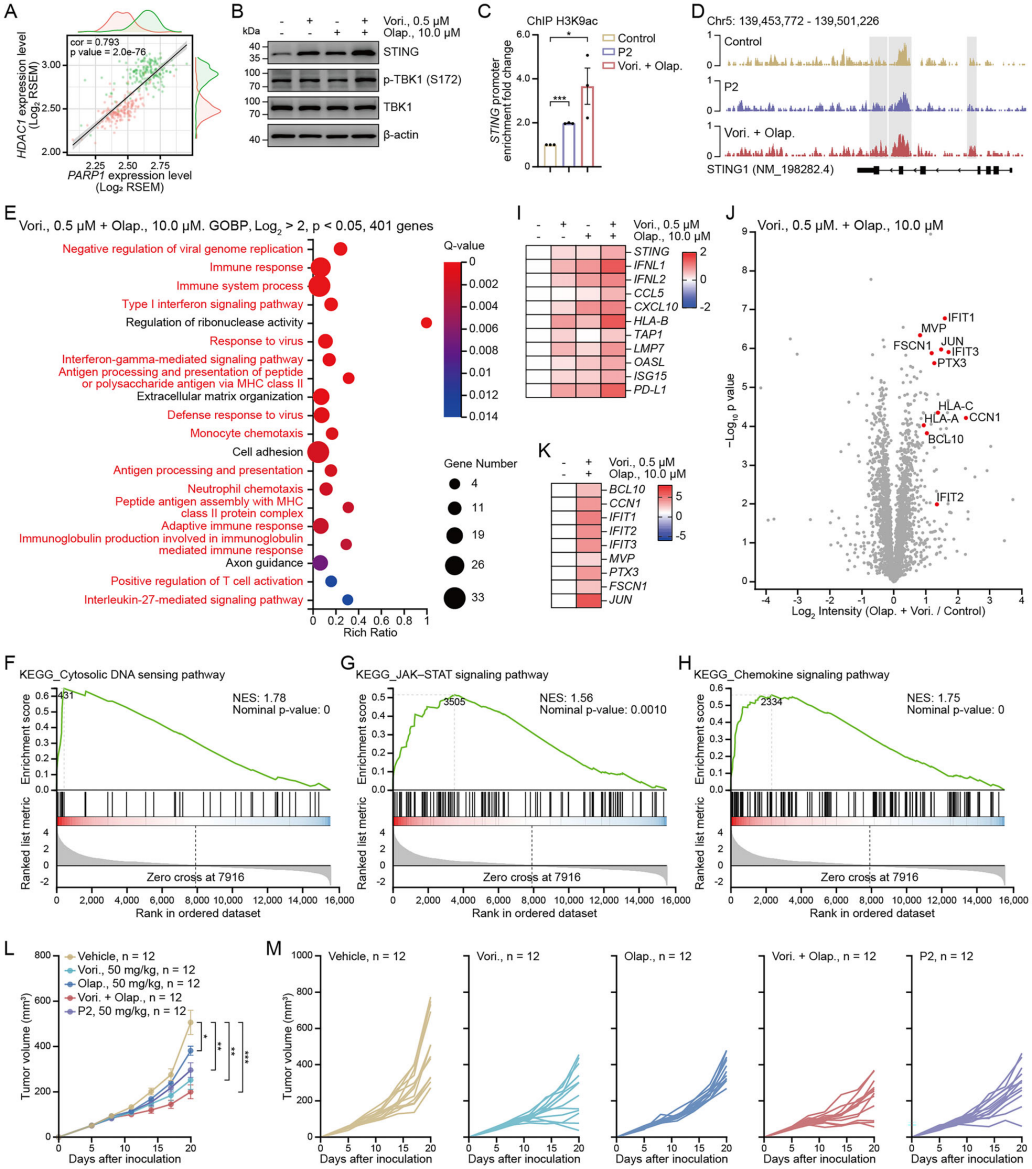
HDAC and PARP inhibitors synergistically activate STING signaling in STING‐defective cells. A) Correlation of HDAC1 and PARP1 expression in pancreatic cancer and normal tissues; *n* = 348. B) STING, p‐TBK1, and TBK1 expression in MIA PaCa‐2 cells treated with Vorinostat (0.5 µm) Olaparib (10.0 µm), or their combination for 48 h; n ≥ 3. C) ChIP analysis of MIA PaCa‐2 cells treated with Vorinostat (0.5 µm) and Olaparib (10.0 µm) in combination, or P2 (5.0 µm) for 48 h; n ≥ 3. D) Gene track visualization of ChIP‐seq data showing promoter acetylation at the STING locus following treatment with Vorinostat (0.5 µm) and Olaparib (10.0 µm) in combination, or P2 (5.0 µm); n ≥ 3. E) GO biological process enrichment analysis on the RNA‐seq data of MIA PaCa‐2 cells treated with Vorinostat (0.5 µm) and Olaparib (10.0 µm) in combination for 48 h; n ≥ 3. F–H) GSEA for gene sets associated with the dsDNA sensing innate immune signaling. I) Heatmap showing RT–qPCR data for indicated genes in MIA PaCa‐2 cells treated with Vorinostat, Olaparib, or their combination for 48 h; n ≥ 3. J) Volcano plot of differentially expressed proteins between the control and combined treatment groups; n ≥ 3. K) Heatmap showing RT–qPCR data for indicated genes in MIA PaCa‐2 cells treated with Vorinostat and Olaparib in combination for 48 h; n ≥ 3. L,M) The tumor growth curves plotted as tumor volume versus time since treatment; *n* = 12. The data are expressed as mean ± SEMs; unpaired student's *t*‐test; ^*^ *p* < 0.05; ^**^ *p* < 0.01; ^***^ *p* < 0.001.

HDACis reinduced STING expression in tumor cells by acetylating the STING promoter. Moreover, HDACis induce “BRCAness” to restore synthetic lethality for improving PARPi efficacy,^[^ ^32^, ^33^ ^]^ which can promote cytosolic DNA accumulation. Accumulation of cytosolic DNA activated the cGAS–STING pathway. We hypothesized that STING‐mediated antitumor response may be amplified by simultaneous treatment with HDACi and PARPi. To test this hypothesis, we first investigated the clinical relevance of this relationship between HDACs and PARPs by analyzing the differences in their expression between tumor and normal tissue in The Cancer Genome Atlas (TCGA) dataset. The expression of HDACs and PARPs was higher in tumor tissues than in normal tissues (Figure S3A,B, Supporting Information). Furthermore, we observed a significant pairwise correlation between HDACs and PARPs (**Figure** **2**A; Figure S3C, Supporting Information). Collectively, these results suggest that HDACs and PARPs are highly involved in tumorigenesis and tumor progression.

To assess whether combined HDAC and PARP inhibition activates the STING pathway in STING‐deficient cells, MIA PaCa‐2 cells were treated with the HDACi Vorinostat, PARPi Olaparib, or both. Notably, the combination treatment markedly increased STING expression and TBK1 phosphorylation compared to those induced by monotherapy or in the control (Figure 2B). Concordantly, ChIP assays revealed enhanced STING transcription and increased H3K9ac enrichment at its promoter following the combination treatment (Figure 2C,D). To investigate the underlying mechanisms, cells treated with a combination of HDACi and PARPi were subjected to RNA sequencing analysis. Gene ontology (GO) enrichment analysis suggested that the combination treatment activated the immune‐ and antiviral‐related signaling pathways, and other major changes in the signaling pathway also focused on the IFN signaling pathways, and antigen processing and presentation (Figure 2E). Gene set enrichment analysis (GSEA) revealed that the genes upregulated by the combination treatment were significantly enriched in the cytosolic DNA‐sensing pathway, JAK–STAT signaling, chemotaxis, cytokine production pathways, T cell immune response, and antigen processing and presentation (Figure 2F–H; Figure S3D–M, Supporting Information). Moreover, real‐time quantitative polymerase chain reaction (RT–qPCR) and proteomics results showed that the combined use of HDACi and PARPi significantly increased STING mRNA levels and the key components of innate immune signaling gene and protein expression such as IFNs, chemokines (CCL5 and CXCL10), antigen processing and presentation (HLAs, TAPs, and LMPs), ISGs (OASL, ISG15, and IFITs), and inflammation‐ or immune‐related pathways (CCN1, PTX3, FSCN1, JUN, and MVP) (Figure 2I–K; Figure S3N, Supporting Information). Overall, these data indicate that cotreatment with HDACi and PARPi synergistically trigger STING signaling in epigenetically *STING*‐silenced cells.

### 2.3 Bifunctional HDAC and PARP Inhibitor Exhibits Potent Antitumor Effect

To evaluate the synergistic antitumor effect of HDAC and PARP inhibition, we knocked down *HDAC1* and *PARP1* in MIA PaCa‐2 cells. RT–qPCR and immunoblotting confirmed effective silencing of each target. While individual knockdown of *HDAC1* or *PARP1* modestly reduced cell proliferation (Figure S4A,B, Supporting Information), simultaneous knockdown resulted in significantly relatively high suppression of cell viability, indicating a synergistic inhibitory effect on tumor cell growth. Colony formation assay also revealed a significant dose‐dependent reduction in the number of cell colonies upon Vorinostat and Olaparib co‐treatment of MIA PaCa‐2 and KP4 cells (Figure S4C, Supporting Information). This combination markedly upregulated apoptosis‐related proteins, indicative of increased cell death (Figure S4D, Supporting Information). Synergy analysis using SynergyFinder demonstrated that Vorinostat dose‐dependently reduced cell viability,^[^ ^35^ ^]^ and this effect was potentiated by increasing Olaparib concentrations (Figure S4E, Supporting Information). Notably, the highest single agent (HSA) synergy score exceeded 10 in MIA PaCa‐2 cells, supporting a synergistic interaction between the two agents (Figure S4F,G, Supporting Information). These findings further support the hypothesis that the dual inhibition of HDAC and PARP exerts a robust synergistic antitumor effect. In vivo experiments further demonstrated that the combined HDAC and PARP inhibition suppressed tumor growth more effectively than that with treatment by either agent alone (Figure 2L,M). Importantly, no histopathological abnormalities were observed in the major organs (Figure S4H, Supporting Information), and the parameters of hepatic, cardiac, and renal functions remained within normal ranges following the combination treatment (Figure S4I–K, Supporting Information), supporting the favorable safety profile of this regimen and its potential for clinical translation.

**Figure 3:**
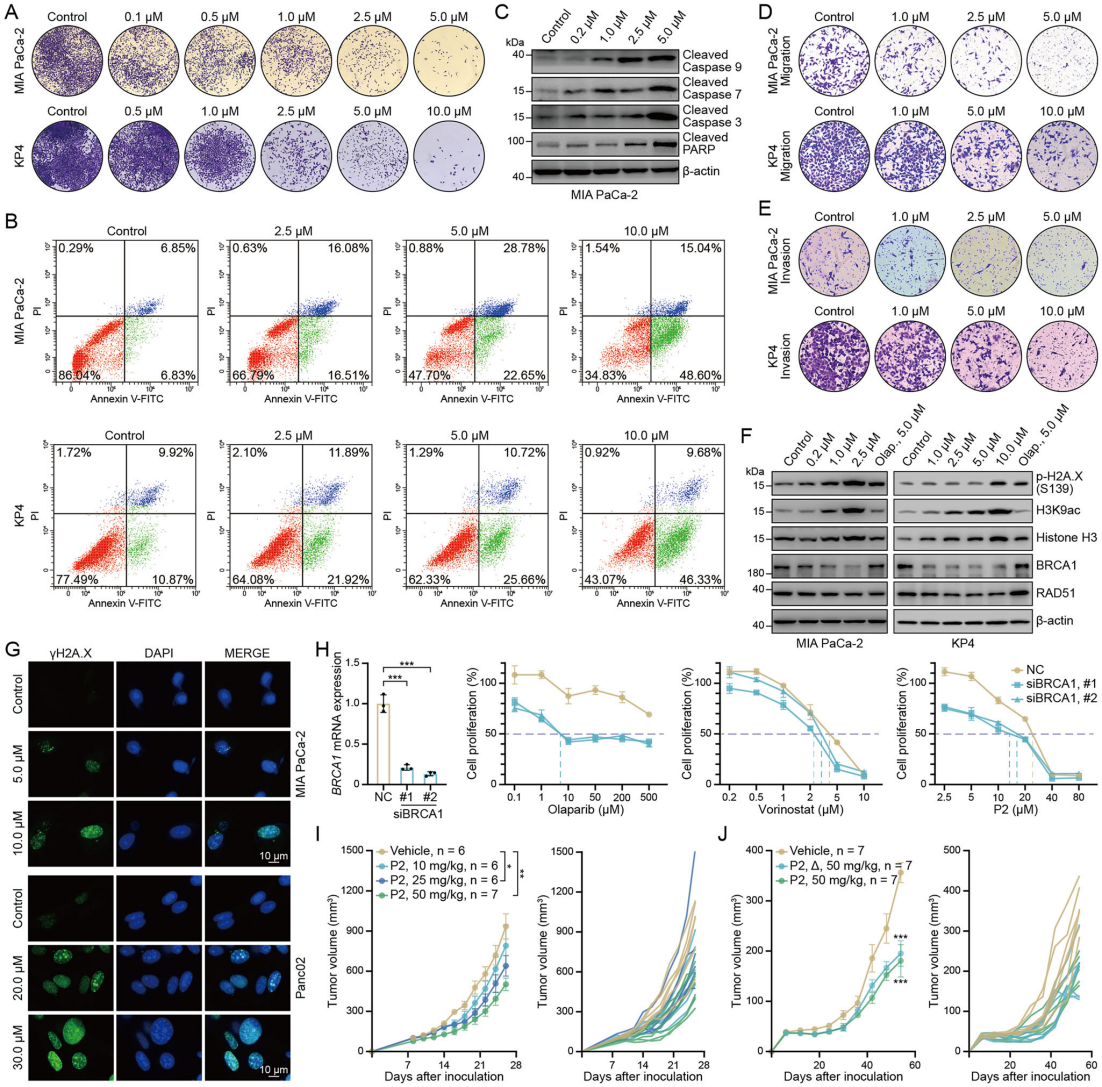
Bifunctional HDAC and PARP inhibitor exhibits a potent antitumor effect. A) P2 inhibited the clonogenicity of pancreatic cancer cells in a dose‐dependent manner. Representative colony at magnification: ×50; n ≥ 3. B) MIA PaCa‐2 and KP4 cells were treated with P2 at indicated concentrations for 48 h, and apoptosis was assessed by flow cytometry; n ≥ 3. C) Apoptosis‐related protein expression levels were analyzed in MIA PaCa‐2 cells treated with P2 at the indicated concentrations for 48 h; n ≥ 3. D,E) Transwell assay to detect the migration and invasion ability of MIA PaCa‐2 and KP4 cells treated with different concentrations of P2 for 48 h; n ≥ 3. F) MIA PaCa‐2 and KP4 cells were treated with P2 at different concentrations for 48 h, and the cell lysates were analyzed by immunoblotting with the indicated antibodies; n ≥ 3. G) MIA PaCa‐2 and Panc02 cells treated with P2 at different concentrations for 48 h, and then subjected to immunofluorescence analysis for γH2A.X; n ≥ 3. H) BRCA1 knockdown MDA‐MB‐157 cells treated with Vorinostat, Olaparib and P2. Cell viability was assessed using the CCK8 assay; n ≥ 3. I,J) MC38 and Panc02 tumor‐bearing mice treated with vehicle or P2 at indicated concentrations. The tumor growth curves plotted as tumor volume versus time since treatment; n ≥ 6. The data are expressed as mean ± SEMs; unpaired student's *t*‐test; ^*^ *p* < 0.05; ^**^ *p* < 0.01; ^***^ *p* < 0.001.

Building on the above findings, we developed a series of small‐molecule precursors with dual inhibitory activities against HDACs and PARPs (Figure S5A, Supporting Information).^[^ ^32^, ^36^ ^]^ We investigated their effects on enzymatic activities and tumor cell proliferation. Compared with the FDA‐approved HDACi Vorinostat and PARPi Olaparib, P1 and P2 exhibited markedly enhanced inhibition of both HDAC and PARP activities (Figure S5B, Supporting Information), and demonstrated relatively high antiproliferative efficacy across a panel of human and murine tumor cell lines (Figure S5C,D, Supporting Information). Given that P2 was more potent than P1 according to a comprehensive analysis, we chose P2 as a representative bifunctional HDAC and PARP inhibitor for evaluating its antitumor effects. Similar to the results obtained using the combination of Vorinostat and Olaparib (Figure S4C, Supporting Information), P2 treatment dose‐dependently reduced the clonogenic capacity of pancreatic cancer cells (**Figure** **3**A). Flow cytometric analysis further confirmed increased apoptosis following P2 exposure (Figure 3B), which was supported by elevated levels of cleaved apoptotic markers revealed by western blot analysis (Figure 3C). In addition, P2 induced G1 phase cell cycle arrest and markedly suppressed migration and invasion of MIA PaCa‐2 and KP4 cells in a dose‐dependent manner (Figure 3D,E; Figure S5E,F, Supporting Information).

As P2 encompassed the PARPi activity, and HDACis induce “BRCAness,” we investigated the effect of P2 on DNA damage responses. P2 treatment dose‐dependently increased the level of phospho‐H2A.X (S139), a canonical marker of DNA double‐strand breaks (Figure 3F), which was corroborated by the accumulation of γH2A.X in nuclear foci as detected via immunofluorescence (Figure 3G). Notably, P2 elevated global H3K9 acetylation while suppressing BRCA1 and RAD51 expression, indicating its HDAC inhibitory activity and the potential of impairing homologous recombination repair (Figure 3F). We also evaluated the effects of HDAC or PARP inhibition by Vorinostat, Olaparib, and P2 in *BRCA1* knockdown MDA‐MB‐157 cells (BRCA1 and BRCA2 wild‐type).^[^ ^37^ ^]^ Downregulation of *BRCA1* expression, as validated using RT–qPCR, enhanced cellular sensitivity to Olaparib and P2, but not to Vorinostat (Figure 3H), suggesting that P2 inhibits both HDAC and PARP, and that the HDAC‐inhibitory activity can induce “BRCAness” for restoring synthetic lethality to improve PARPi efficacy. Finally, we explored the in vivo antitumor efficacy of P2 in a syngeneic tumor model of mice. Consistent with the in vitro results, P2 significantly delayed MC38 (a murine colorectal cancer model) tumor growth in a dose‐dependent manner (Figures 2L,M and 3I). Similar results were observed in the P2‐treated Panc02 subcutaneous model (a murine pancreatic cancer model) (Figure 3J). Collectively, these results indicate that P2 exerts potent antitumor effects and induces DNA damage in tumor cells by inhibiting both HDAC and PARP.

### 2.4 P2 Can Restore and Activate STING Signaling in STING‐Silenced Tumor Cells

**Figure 4:**
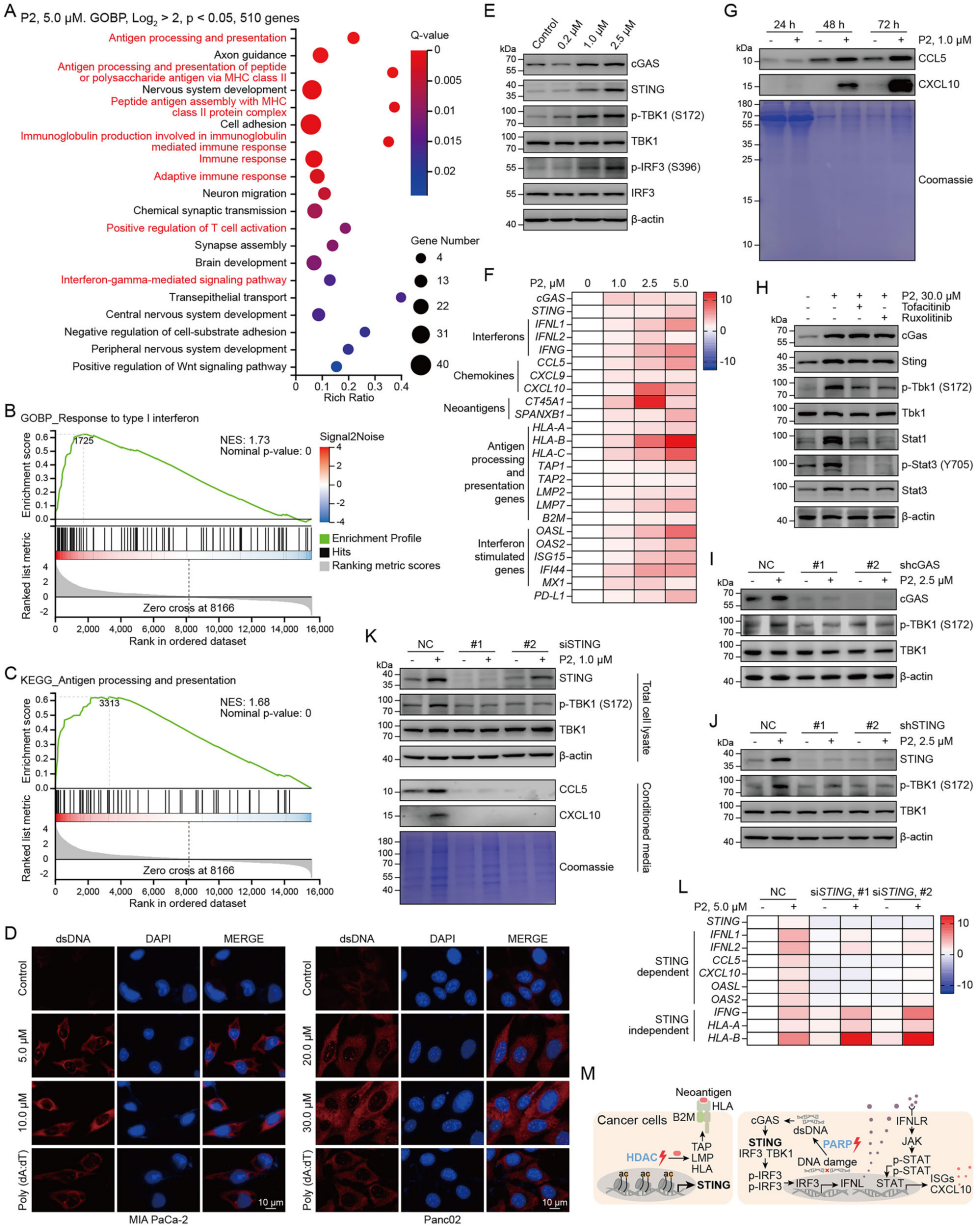
P2 can restore and activate STING signaling in STING‐silenced tumor cells. A) GO biological process enrichment analysis on the RNA‐seq results of MIA PaCa‐2 cells treated with 5.0 µm P2 for 48 h; n ≥ 3. B,C) GSEA for gene sets associated with the IFN‐ and antigen presentation‐related signaling pathways. D) Representative confocal images of immunofluorescence analysis using anti‐dsDNA antibody (red) after treatment with P2 for 48 h; n ≥ 3. E) Expression of cGAS–STING signaling pathway‐related proteins in MIA PaCa‐2 cells treated with P2 for 48 h, analyzed using immunoblotting; n ≥ 3. F) Heatmap showing RT–qPCR data for indicated genes in MIA PaCa‐2 cells treated with P2 for 48 h; n ≥ 3. G) Analysis of secreted chemokines in P2‐treated MIA PaCa‐2 cells via immunoblotting; n ≥ 3. H) Panc02 cells were pretreated with JAK inhibitors (Tofacitinib and Ruxolitinib) and stimulated with P2 for 48 h. The levels of cGAS–STING signaling pathway‐related proteins were analyzed using immunoblotting; n ≥ 3. I,J) p‐TBK1 levels in *cGAS* and STING knockdown MIA PaCa‐2 cells were treated with P2; n ≥ 3. L) Heatmap showing RT–qPCR data for indicated genes in STING knockdown MIA PaCa‐2 and control cells treated with P2 for 48 h; n ≥ 3. M) Schematic of a bifunctional HDAC and PARP inhibitor remodels the tumor microenvironment.

Next, we investigated whether P2‐induced histone acetylation and accumulation of damaged DNA contributed to re‐expression and activation of STING signaling in STING‐deficient MIA PaCa‐2 cells. GO enrichment analysis revealed that P2 treatment significantly upregulated the genes involved in antigen processing and presentation, immune response, and the IFN signaling pathways (**Figure** **4**A), which were further validated using GSEA (Figure 4B–C; Figure S6, Supporting Information). P2 treatment significantly increased cytosolic dsDNA levels (Figure 4D). Importantly, similar to that by FDA‐approved HDACis, P2 treatment effectively restored STING expression (Figure 4E; Figure S7A, Supporting Information). Furthermore, P2 dose‐dependently increased the phosphorylation of TBK1 (S172) and IRF3 (S396) (Figure 4E; Figure S7A,B). We also observed increased production of CCL5 and CXCL10 in a time‐ and dose‐dependent manner (Figure 4F,G; Figure S7C,D, Supporting Information). These changes were confirmed at the mRNA level using RT–qPCR (Figure 4F; Figure S7C, Supporting Information). In addition, P2 treatment dose‐dependently induced IFNs, chemokines, neoantigen‐related genes, antigen‐processing genes, and ISGs (Figure 4F). These results indicate that P2 restores STING expression and functionally activates tumor‐intrinsic STING signaling.

IFNs activate cell‐intrinsic signaling by engaging the IFN receptor and subsequently activating the JAK–STAT pathway, leading to the induction of ISGs and proinflammatory chemokines.^[^ ^7^, ^8^ ^]^ To investigate whether JAK–STAT signaling is required for P2‐mediated activation of STING signaling, we treated MIA PaCa‐2 cells with the JAK inhibitors Tofacitinib and Ruxolitinib. Both inhibitors effectively suppressed P2‐induced phosphorylation of TBK1 and STAT3 (Figure 4H), suggesting that JAK activity is essential for activation of the STING pathway induced by P2. To further confirm that P2‐induced TBK1 phosphorylation and CCL5/CXCL10 production are mediated specifically through the cGAS–STING signaling axis, rather than through off‐target effects, we knocked down *cGAS* or *STING*. Knockdown of either component markedly attenuated P2‐induced TBK1 phosphorylation (Figure 4I,J; Figure S7E,F, Supporting Information). Similarly, STING depletion abrogated P2‐mediated secretion of CCL5 and CXCL10 (Figure 4K). Consistent with these findings, STING knockdown reduced the mRNA expression of CCL5, CXCL10, and IFNs (Figure 4L). Notably, STING silencing did not impair P2‐induced upregulation of *HLA* transcripts, suggesting that the induction of antigen‐presenting genes may occur via HDAC inhibition independent of STING. Collectively, these results indicate that P2 functions as a potent bifunctional HDAC and PARP inhibitor, capable of restoring and activating STING signaling in STING‐deficient tumor cells (Figure 4M).

### 2.5 P2 Enhances Antitumor Immunity by Recruiting T Cells and Potentiates Anti‐PD‐L1 Efficacy in Syngeneic Models

**Figure 5:**
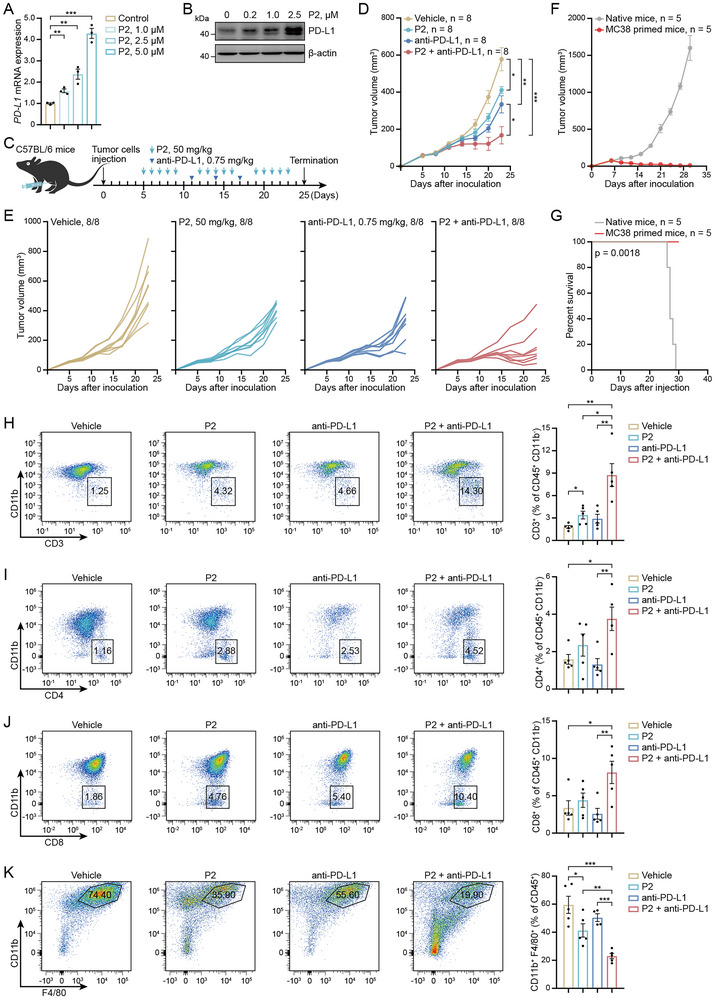
P2 enhances antitumor immunity by recruiting T cells and potentiates anti‐PD‐L1 efficacy in syngeneic models. A,B) PD‐L1 expression in MIA PaCa‐2 cells treated with P2; n ≥ 3. C) Schematic of P2 and anti‐PD‐L1 treatments in MC38 tumor‐bearing mice. D,E) Tumor volumes of MC38 tumor‐bearing mice treated with vehicle, P2, anti‐PD‐L1, or a combination of P2 and anti‐PD‐L1; *n* = 8. F,G) Tumor volumes and survival curves of naïve or rechallenged MC38 tumor‐free mice; *n* = 5. H–K) Infiltration of CD3^+^, CD4^+^, and CD8^+^ T cells, and macrophages in MC38 tumors detected using flow cytometry; n ≥ 4. The data are expressed as mean ± SEMs; unpaired student's *t*‐test; ^*^ *p* < 0.05; ^**^ *p* < 0.01; ^***^ *p* < 0.001.

Consistent with previous reports indicating that treatment with HDACis or PARPis can upregulate the immune checkpoint ligand PD‐L1 in tumor cells,^[^ ^15^, ^22^, ^38^ ^]^ P2 treatment markedly increased PD‐L1 expression at the mRNA and protein levels in a dose‐dependent manner (**Figure** **5**A,B). Therefore, we evaluated whether P2 treatment could sensitize tumors to ICB therapy by intraperitoneally injecting mice with an anti‐PD‐L1 antibody (Figure 5C). As previously reported,^[^ ^39^ ^]^ ICB therapy using an anti‐PD‐L1 antibody reduced tumor growth (Figure 5D,E). The combination of anti‐PD‐L1 with P2 resulted in rapid tumor regression compared to that in mice treated with either P2 or anti‐PD‐L1, and in some mice, the tumor was completely cleared (Figure 5D,E). After two months of follow‐up, all tumor‐free mice were re‐challenged with five times the number of initially injected tumor cells on the opposite flank. Notably, rechallenged tumors were fully rejected, with longer survival than that of age‐matched naïve mice (Figure 5F,G), demonstrating that these convalescent mice established robust and durable adaptive immune memory.

To clarify the mechanisms underlying the tumor‐suppressive effects of P2 alone or in combination with anti‐PD‐L1, we analyzed the tumor immune profiles using multicolor flow cytometry (Figure S8A,B, Supporting Information). The combination of P2 and PD‐L1 blockade markedly increased immune cell (CD45^+^) infiltration in the MC38 model (Figure S8C, Supporting Information). P2 alone significantly increased infiltration of intratumoral CD3^+^ T cells, and CD4^+^ and CD8^+^ T cells (Figure 5H–J), and reduced the proportion of macrophages (F4/80^+^ CD11b^+^), whereas anti‐PD‐L1 monotherapy did not (Figure 5K), suggesting a relative decrease in immunosuppressive myeloid cells following P2 treatment. The addition of an anti‐PD‐L1 antibody further reinforced these trends, showing significant increases in CD3^+^, CD4^+^, and CD8^+^ T cells and a significant decrease in macrophages (Figure 5H–K). These findings may explain why the combination of P2 and anti‐PD‐L1 antibody demonstrates higher antitumor efficacy than does either treatment alone.

### 2.6 Paracrine Signaling from P2‐Treated Tumor Cells Induces DC Maturation and Activation

**Figure 6:**
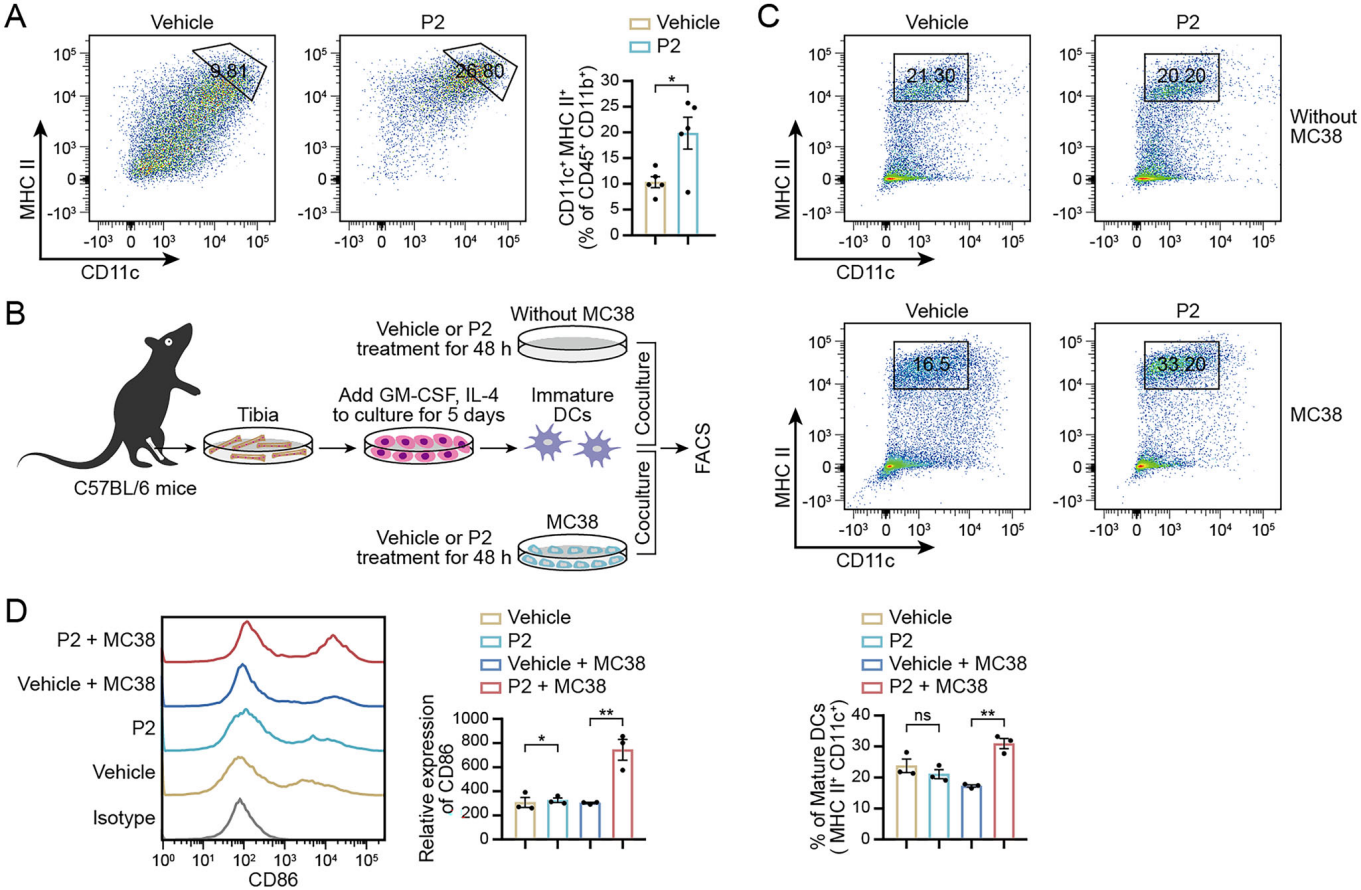
Paracrine signaling from P2‐treated tumor cells induces DC maturation and activation. A) Infiltration of DCs in MC38 tumors detected using flow cytometry; *n* = 5. B) Schematic of co‐culturing immature BMDCs with or without MC38 in the presence of DMSO or P2 for 48 h. C) Representative flow cytometric plots showing frequencies of mature DCs (CD11c^+^ MHC II^high^); *n* = 3. D) Representative histograms and mean fluorescence intensity of CD86 expression on BMDCs; *n* = 3. The data are expressed as mean ± SEMs; unpaired student's *t*‐test; ns, not significant; ^*^ *p* < 0.05; ^**^ *p* < 0.01.

DCs within the tumor microenvironment play a key role in capturing tumor‐derived DNA primarily through a STING‐dependent mechanism, promoting the presentation of tumor‐specific antigens, and subsequently triggering the activation of cytotoxic T lymphocytes. We assessed the changes in the proportion of intratumoral DCs in MC38 tumors. P2 treatment substantially elevated the proportion of mature DCs and significantly upregulated major histocompatibility complex class II (MHC II) expression (**Figure** **6**A), suggesting enhanced antigen‐presenting capability of these tumor‐associated DCs in response to P2. Importantly, DC maturation and activation are essential for initiating an immune response and activating subsequent adaptive antitumor immunity.^[^ ^40^ ^]^

To determine whether the observed DC maturation and activation results from a direct effect by P2 or P2‐treated tumor cell‐mediated paracrine activation, we differentiated and isolated immature mouse bone marrow‐derived DCs (BMDCs) on day 5 and cultured them with or without MC38 in the presence of dimethyl sulfoxide (DMSO) or P2; DC maturation was then assessed via flow cytometry (Figure 6B). Co‐culture with P2‐treated MC38 cells induced maturation and activation of BMDCs more significantly than that in BMDCs co‐cultured with DMSO‐treated MC38 cells (Figure 6C). Furthermore, co‐culture with MC38 cells significantly increased the expression of the co‐stimulatory molecule CD86 in BMDCs in response to P2 treatment (Figure 6D). CD86 is essential for delivering secondary activation signals to T cells,^[^ ^9^ ^]^ indicating that P2 enhances DC activation and promotes subsequent antitumor immune responses. Notably, P2 treatment alone did not significantly induce DC maturation or activation, when not co‐cultured with MC38 tumor cells (Figure 6C,D), suggesting that P2 exerted its effects on DCs in a paracrine manner. These results indicate that cytosolic dsDNA and IFNs released from P2‐treated tumor cells contribute to the subsequent maturation and activation of DCs, highlighting a tumor‐intrinsic mechanism that enhances antitumor immunity through crosstalk with the immune microenvironment.

### 2.7 Intact Intratumoral STING Pathway is Required for P2‐Induced Antitumor Immune Response

**Figure 7:**
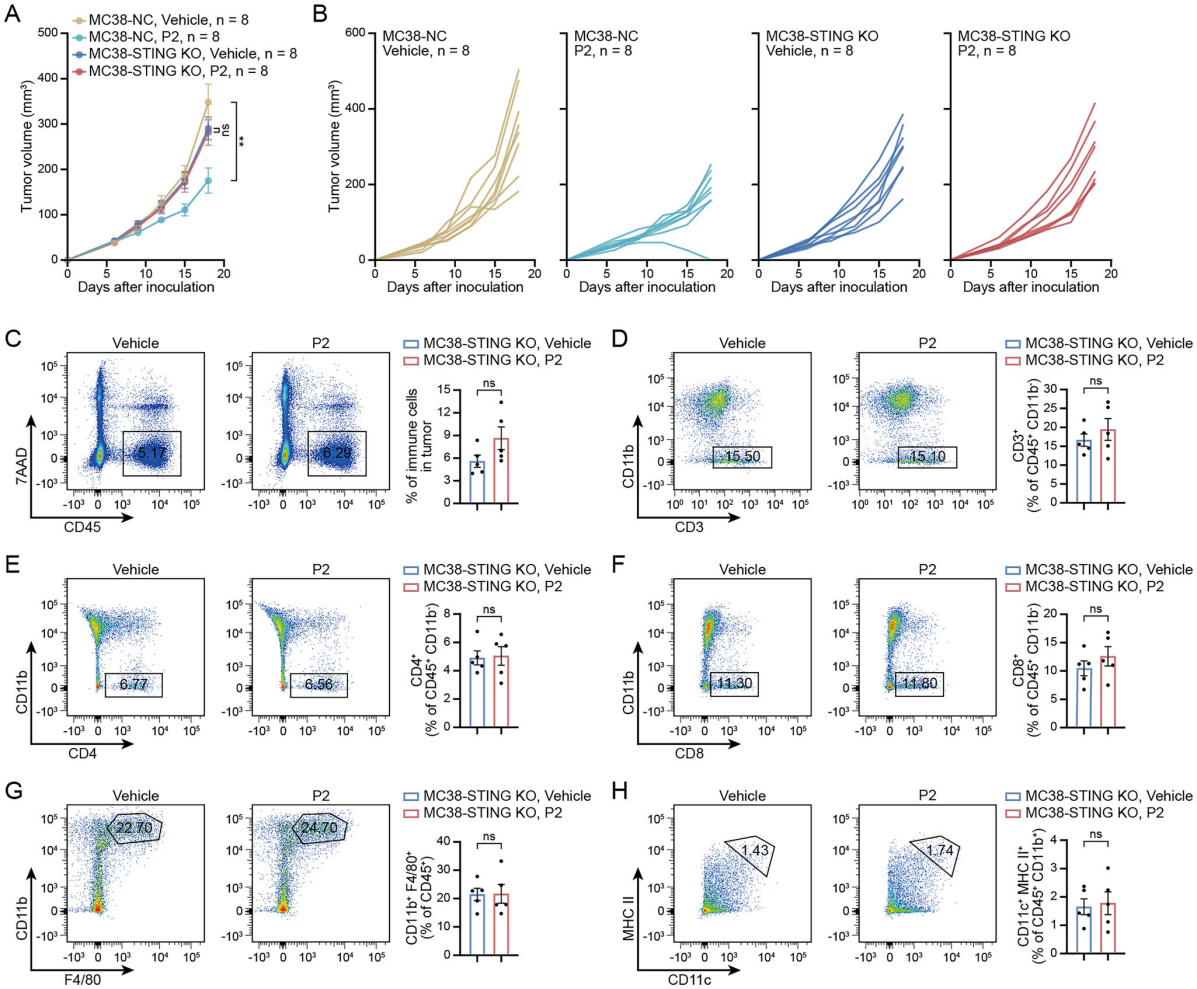
Intact intratumoral STING pathway is required for P2‐induced antitumor immune response. A,B) The tumor volumes of MC38 wild‐type and STING KO tumor‐bearing mice treated with vehicle or P2; *n* = 8. C–H) Infiltration of CD45^+^, CD3^+^, CD4^+^, and CD8^+^ T cells, macrophages, and DCs in MC38 STING KO tumors detected using flow cytometry; *n* = 5. The data are expressed as mean ± SEMs; unpaired student's *t*‐test; ns, not significant; ^**^ *p* < 0.01.

To directly evaluate the necessity of the STING pathway activation in tumor cells for P2‐induced immune cell recruitment in vivo, we used CRISPR/Cas9 to deplete STING in MC38 cells and subcutaneously injected these tumor cells into syngeneic mice. The efficiency of depletion was determined using immunoblot analysis, and knockout (KO) of STING impaired P2‐induced phosphorylation of TBK1 (Figure S7F, Supporting Information). Consistent with our previous results, P2 significantly inhibited tumor growth in the STING wild‐type control (**Figure** **7**A,B). Notably, the antitumor efficacy of P2 was completely abolished in STING KO mice, suggesting that STING deficiency in tumor cells impairs the efficacy of P2 in vivo.

Next, we performed flow cytometric analysis of the collected STING KO tumors treated with vehicle or P2 for 3 weeks (gating strategies were same as that in Figure S8A,B, Supporting Information). Tumor tissues maintained low levels of STING (Figure S8D, Supporting Information), which differed from tumor cells, and might have been contributed by tumor‐infiltrating immune cells. In contrast to our previous findings in STING wild‐type tumor models, P2 treatment did not significantly alter the frequency of intratumoral immune (CD45^+^) and total T (CD3^+^) cell infiltration (Figure 7C,D), and CD4^+^ and CD8^+^ T cells (Figure 7E,F) in STING KO tumors. Furthermore, P2‐induced reduction of macrophages and increased DC maturation were also abolished when STING was depleted (Figure 7G,H), indicating that STING depletion impairs lymphocyte recruitment in response to P2. Overall, these results confirm the vital role of the tumor cell‐intrinsic STING pathway in P2‐mediated antitumor immune response.

## 3 Discussion

cGAS–STING signaling serves as a key regulator of the cytosolic DNA‐sensing pathway, driving the activation of type I and III IFNs in the host immune response. As its role in cancer immunology has been elucidated, pharmacological STING agonists that induce antitumor immunity have been investigated.^[^ ^41^, ^42^ ^]^ However, numerous cancers have developed strategies for evading innate immune surveillance, particularly by suppressing the cGAS–STING pathway. cGAS and/or STING deficiency has been reported in multiple tumor types.^[^ ^23^, ^28^ ^]^ Notably, > 20% advanced colorectal cancers show complete loss of STING expression,^[^ ^43^ ^]^ potentially limiting the therapeutic efficacy of STING agonist‐based immunotherapies. In this study, we identified frequent loss of STING expression in pancreatic tumor tissues and cell lines, which was associated with impaired innate immune signaling. This suppression was selective for STING, as cGAS levels remained largely unaffected. In several instances, STING deficiency arises not from genetic alterations, such as mutations or deletions, but rather from epigenetic silencing of STING expression.^[^ ^26^, ^28^, ^43^, ^44^ ^]^ Our findings demonstrate that HDAC inhibition effectively restores STING signaling in pancreatic cancers through epigenetic reprogramming and reveal that HDACis effectively restore STING expression in pancreatic and breast cancer cells, but not in colorectal cancer cells, in which STING is already expressed, suggesting tumor‐specific vulnerability. These observations highlight a tumor‐specific mechanism of STING silencing and underscore the importance of tumor context in designing epigenetic strategies for restoring STING‐mediated innate immunity.

Moreover, HDACis downregulate key DNA damage‐repair proteins, including BRCA1, BRCA2, and RAD51, which have been validated in various cancer types, and HDACi treatment sensitizes cancer cells to the lethal effects of PARPis.^[^ ^32^, ^45^ ^]^ Building on these findings, we developed bifunctional HDAC and PARP inhibitors, with the lead compound P2 effectively restoring STING signaling and triggering a robust tumor‐intrinsic immune response. In addition to its immunomodulatory effects, P2 downregulated key homologous recombination proteins, including BRCA1 and RAD51, leading to impaired DNA repair and increased γH2A.X levels. These features are indicative of a “BRCAness” phenotype. The resulting DNA damage promoted cytosolic accumulation of dsDNA, which in turn activated the cGAS–STING pathway. Therefore, P2 exerts dual effects by inducing synthetic lethality and restoring STING‐mediated innate immune activation in tumor cells. These findings underscore the therapeutic potential of dual‐targeting strategies for restoring innate immune signaling and overcoming immune evasion in STING‐deficient tumors. Co‐administration of HDAC and PARP inhibitors can synergistically reduce BRCA1 expression in prostate cancer^[^ ^33^ ^]^ or induce DNA damage in Ewing sarcoma models,^[^ ^45^ ^]^ such studies have focused on synthetic lethality and cell cycle arrest, with limited attention to immune modulation. Our findings demonstrated that dual inhibition of HDAC and PARP modulated STING‐mediated innate immunity, highlighting a novel immunotherapeutic strategy.^[^ ^32^ ^]^

The activation of tumor‐intrinsic STING signaling leads to IFN production, which facilitates the recruitment of DCs, T cells, and macrophages to the tumor microenvironment, thereby promoting MHC expression and enhancing tumor antigen presentation.^[^ ^6^ ^]^ Our findings indicated that P2 promoted intratumoral infiltration of CD4^+^ and CD8^+^ T cells, and enhanced the recruitment of DCs with potent antigen‐presenting capacity within the tumor microenvironment. These efficacies of P2 were abolished by STING KO in tumor cells, suggesting the role of STING signaling activation in the P2‐mediated antitumor immune response. In addition to restoring STING‐dependent innate immune signaling, P2 enhanced the expression of antigen presentation–related genes through a STING‐independent mechanism. This was supported by sustained upregulation of HLA‐A and HLA‐B in STING‐deficient cells. This effect is likely attributable to the HDAC‐inhibitory activity of P2 and is consistent with those of previous studies demonstrating that HDAC inhibition can epigenetically upregulate the expression of MHC class I genes.^[^ ^46^, ^47^ ^]^ These findings suggest that P2 exerts dual immunomodulatory effects by reactivating innate immunity and promoting tumor antigen presentation, which together may expand its therapeutic potential, particularly in tumors with partial or complete loss of STING expression. Moreover, our data demonstrated that P2 significantly increased PD‐L1 expression in tumor cells, and that P2 therapy enhanced the antitumor immune response of PD‐L1 blockade. P2 in combination with ICB resulted in durable tumor regression followed by the development of immune memory, as rechallenged mice displayed complete and durable tumor rejection without the need for additional treatment.

In summary, we have characterized a bifunctional HDAC and PARP inhibitor that restores STING expression and induces defects in the DNA repair machinery in cancer cells. Considering the potential toxicities associated with HDACi and PARPi monotherapies, and the risk of cumulative toxicities from sequential combination therapies, developing drugs with bifunctional functions is crucial. Our findings offer a strong scientific rationale for designing novel bifunctional HDACi and PARPi for reconstituting STING signaling in STING‐silenced tumors, with no evidence of toxicity. Furthermore, the combination of bifunctional HDAC and PARP inhibitors with ICB therapy can enhance antitumor immunity, providing a new approach for boosting antitumor immunity.

## 4 Experimental Section

Detailed Experimental Sectionare available in Supporting Information Appendix. This includes the following: chemicals, cell lines and cell culture, cell viability assay, colony forming assay, wound healing assay, transwell invasion assay, western blot analysis, immunofluorescence analysis, multiplex immunofluorescence staining, Poly(I:C), Poly(dG:dC) and small‐interfering RNA (siRNA) transfections, lentivirus production and gene knockdown by short‐hairpin RNA (shRNA), RNA isolation and real‐time quantitative polymerase chain reaction (RT–qPCR), RNA sequencing (RNA‐seq) analysis, proteomics analysis, ChIP analysis, bone marrow‐derived dendritic cells (BMDCs) isolation and activation assay, animal experiments, hematoxylin and eosin (HE) staining, inhibitor toxicity analysis, flow cytometry analysis, bioinformatics analysis, and statistical analysis.

### Ethics Approval and Consent to Participate

The collection of clinical specimens in this study was approved by the Ethics Committee of Outdo Biotech. Informed consent was obtained from all participants. All animal procedures were approved by the Institutional Animal Care and Use Committee at the Shenzhen University (Approval Number: AEWC‐202300007).

## Conflict of Interest

The authors declare no conflict of interest.

## Author Contributions

C.M. and W.F. contributed equally to this work. Y.J. and B.C. conceived the study, designed and supervised the experiments, and revised the manuscript. C.M., W.F., and F.Y. performed the animal study. C.M., W.F., J.L., F.Y., and W.L. performed the cell experiments. Z.S. and Z.Y. designed and synthesized the compounds. C.M., W.F., L.L., Q.L., and B.C. analyzed the data. C.M., W.F., and B.C. drafted the manuscript. All authors contributed to the data acquisition and interpretation. All authors reviewed and approved the manuscript.

## Supporting information



Supporting Information

## Data Availability

The data that support the findings of this study are available from the corresponding author upon reasonable request.
