# Supplementary material for: Targeting HDAC and PARP Enhances STING‐Dependent Antitumor Immunity in STING‐Deficient Tumor
Source: Adv Sci (Weinh). 2025 Aug 11;12(41):e07904. doi: 10.1002/advs.202507904 (PMC12591105; doi:10.1002/advs.202507904)
Supplement: Supplementary file 1 — Supporting Information [file ADVS-12-e07904-s001.docx]

# Supporting Information

**Targeting HDAC and PARP Enhances STING-Dependent Antitumor Immunity in STING-Deficient Tumor**

Chengzhou Mao ^1,#^, Weiwen Fan ^2,#,5^, Jiaqi Liu ^3^, Fangzhou Yang ^2^, Wenkai Li ^2^, Lulu Li ^4^, Zhichao Shi ^3^, Qinyuan Li ^4^, Zigao Yuan ^4^, Yuyang Jiang ^2,3,4^, Bizhu Chu ^2,*^

^1^ Department of Anatomy and Histology, Shenzhen University Medical School, Shenzhen University, Shenzhen 518055, China

^2^ Guangdong Provincial Key Laboratory of Chinese Medicine Ingredients and Gut Microbiomics, School of Pharmacy, Shenzhen University Medical School, Shenzhen University, Shenzhen 518055, China

^3^ Institute of Biomedical Health Technology and Engineering, Shenzhen Bay Laboratory, Shenzhen 518132, China

^4^ State Key Laboratory of Chemical Oncogenomics, Tsinghua Shenzhen International Graduate School, Shenzhen 518055, China

^5^ Present address: Department of Pharmacy, The Second Hospital of Longyan, Longyan 364099, China

^#^ C.Z. Mao and W.W. Fan contributed equally to this work.

**Corresponding Author:** Bizhu Chu, Shenzhen University, No. 1066, Xueyuan Road, Nanshan District, Shenzhen 518055, Guangdong, China. E-mail: chubz@szu.edu.cn. Phone number: +86-755-26912152; +86 18718698300.

# Supplementary Figures


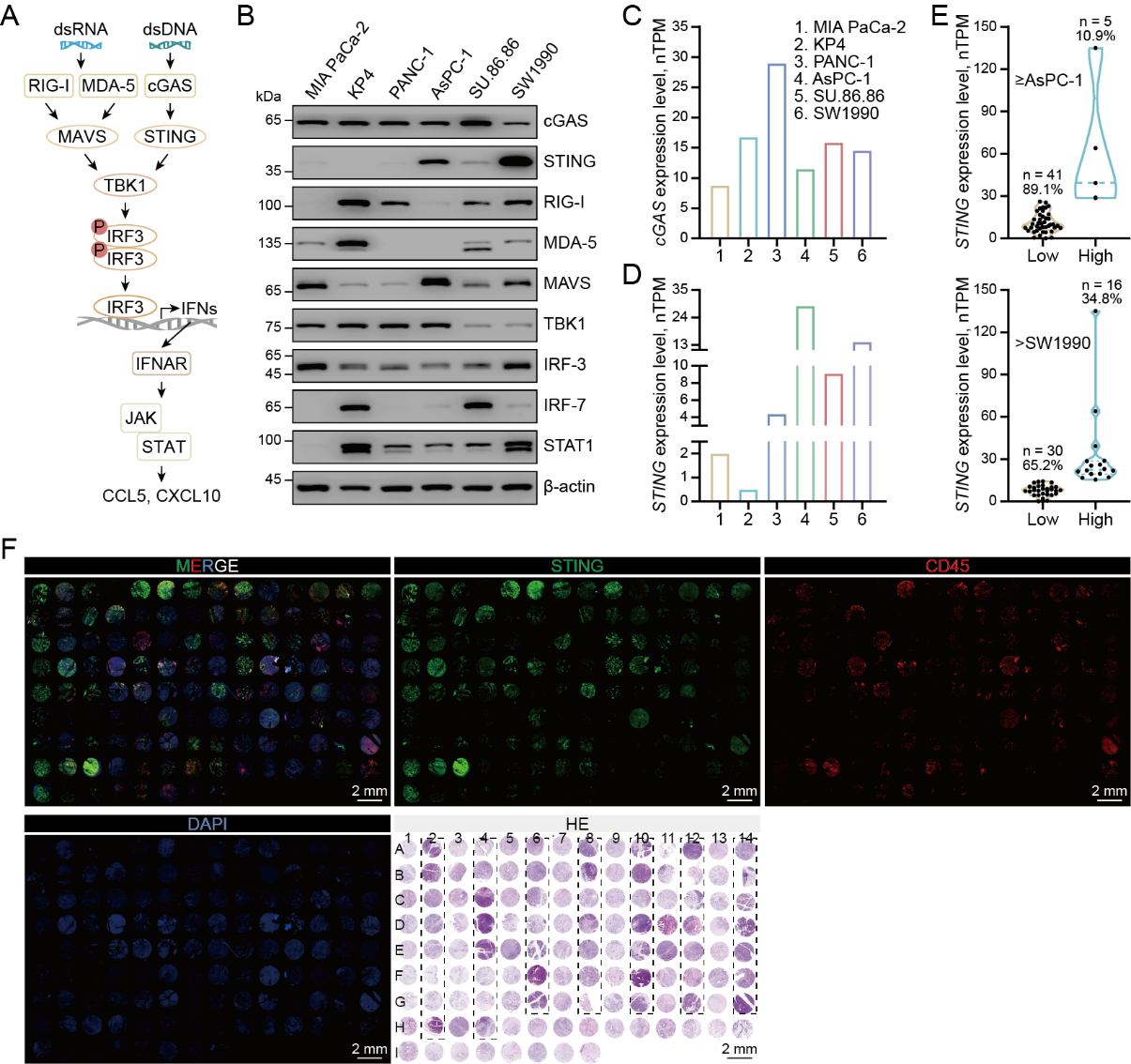


**Figure S1.** STING expression is frequently downregulated in both pancreatic cancer tissues and cell lines. (A) Schematic representation of cell-intrinsic innate immune sensing pathways for cytosolic dsRNA and dsDNA, leading to the induction of IFNs and downstream chemokine production. (B) Expression of proteins related to dsRNA and dsDNA sensing pathways in pancreatic cancer cell lines; n ≥ 3. (C–D) mRNA expression levels of cGAS and STING in pancreatic cancer cell lines extracted from the HPA database. (E) STING expression levels in pancreatic cancer cell lines from the HPA database, with AsPC-1 and SW1990 serving as high-expression references; n = 46. (F) Multiplex immunofluorescence of pancreatic cancer tissue microarray showing STING (green), CD45 (red), and nuclei (DAPI, blue). Dashed boxes mark adjacent non-tumor regions based on corresponding HE staining; n = 69.


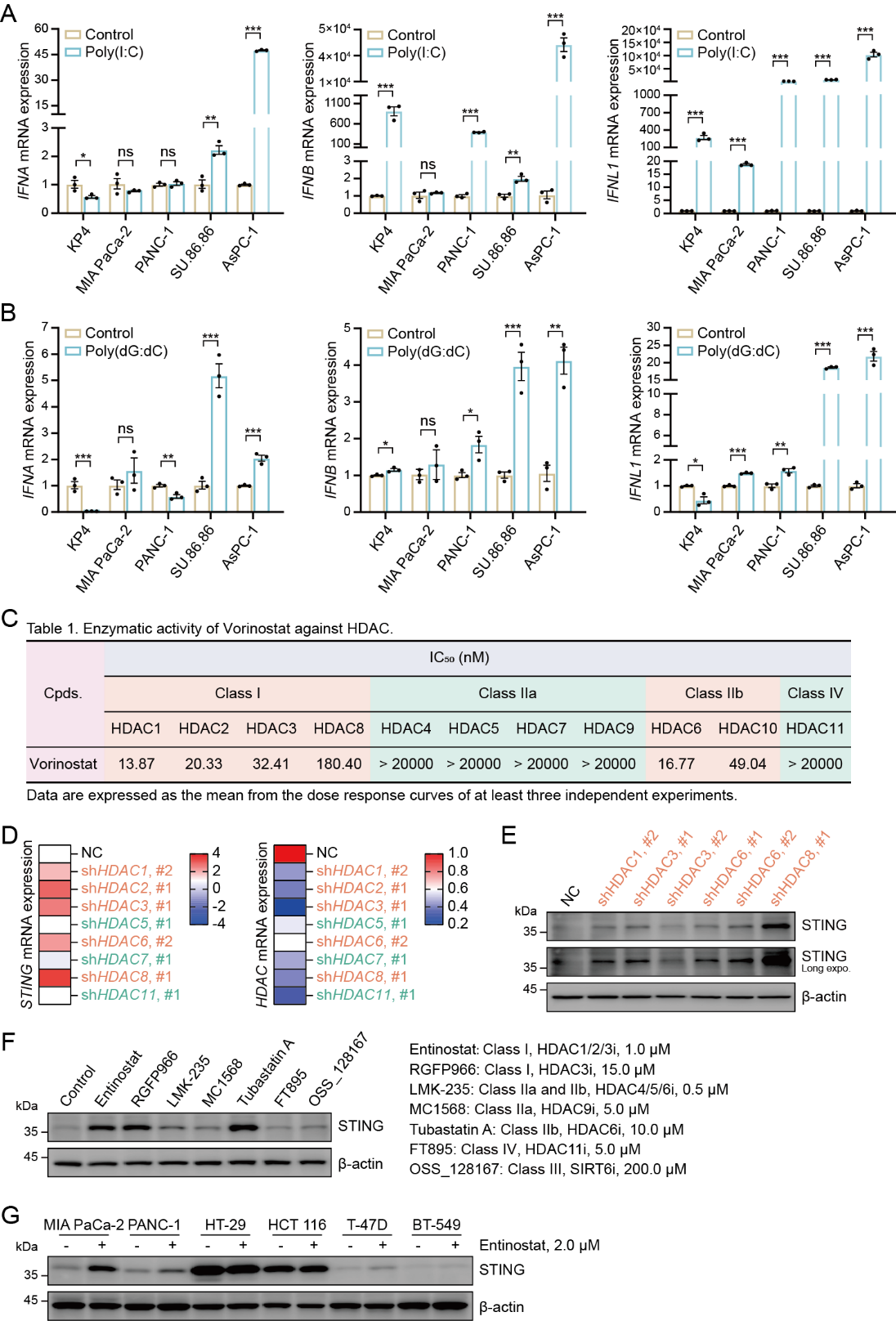


**Figure S2.** HDAC suppressed tumor-intrinsic innate immune signaling via binding and deacetylating STING gene promoter. (A) Type Ⅰ and Ⅲ IFNs transcript levels were analyzed in pancreatic cancer cells using RT–qPCR after transfection with 1 μg/mL Poly(I:C); n ≥ 3. (B) Type Ⅰ and Ⅲ IFNs transcript levels in pancreatic cancer cells transfection with 1 μg/mL Poly(dG:dC); n ≥ 3. (C) Enzymatic activity of Vorinostat against HDACs; n ≥ 3. (D) Heatmap showing RT–qPCR data for *STING* and corresponding *HDACs* in MIA PaCa-2 negative control (NC) cells and a series of cell lines with stable knockdown of HDACs; n ≥ 3. (E) STING expression levels were analyzed in MIA PaCa-2 NC cells and a series of cell lines with stable knockdown of HDACs; n ≥ 3. (F) MIA PaCa-2 cells treated with isoform-specific HDAC inhibitors for 48 h, followed by analysis of STING expression via immunoblotting; n ≥ 3. (G) The pancreatic, colorectal and breast cancer cell lines treated with HDAC inhibitors for 48 h and were analyzed by immunoblotting; n ≥ 3. The data are expressed as mean ± SEMs; unpaired student’s *t*-test; ns, not significant; **p* < 0.05; ***p* < 0.01; ****p* < 0.001.


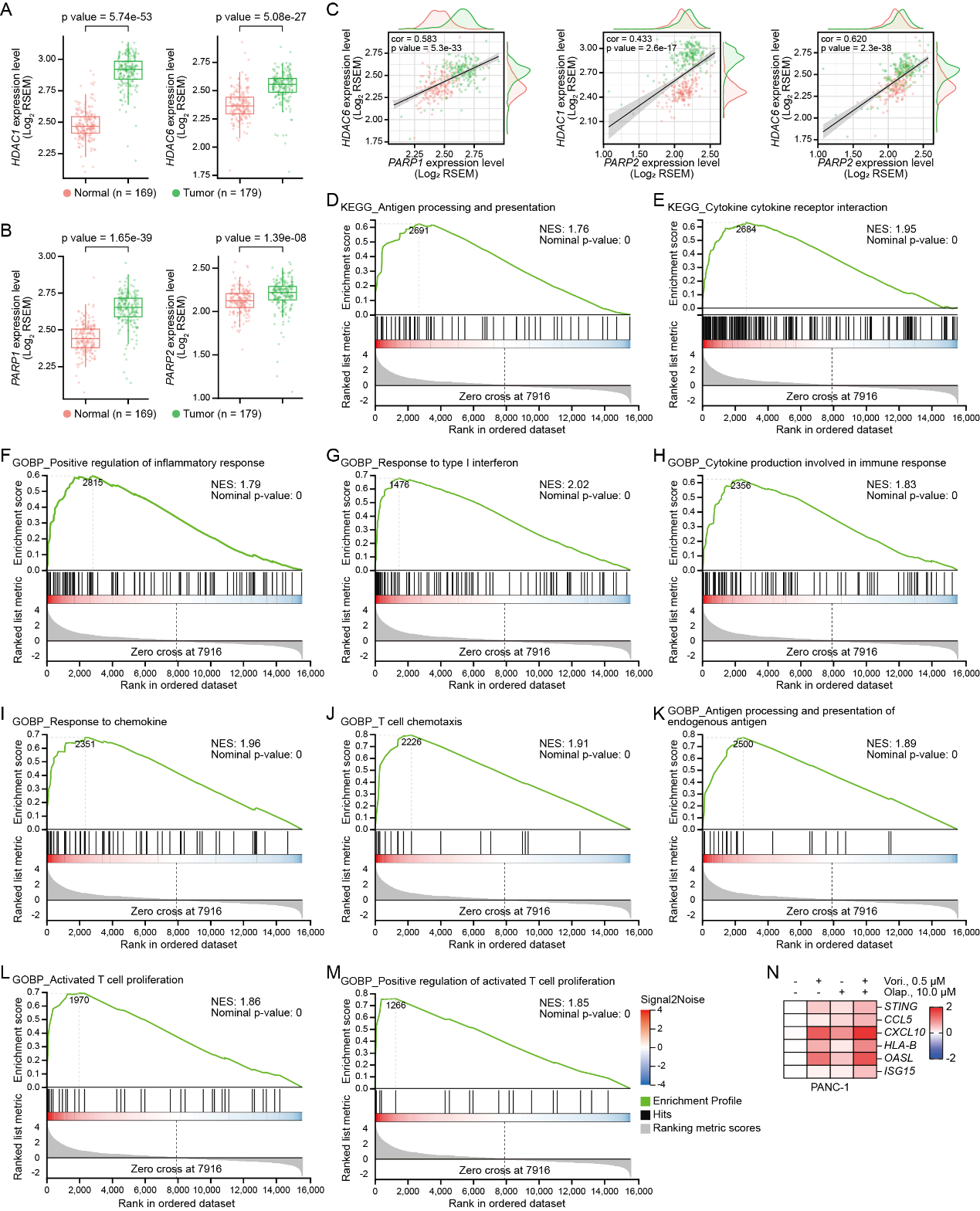


**Figure S3.** HDAC and PARP inhibitors synergistically activate STING signaling in STING-defective cells. (A) Expression levels of HDAC1 and HDAC6 in normal patients comparing with tumor patients; n = 348. (B) Expression levels of PARP1 and PARP2 in normal patients comparing with tumor patients; n = 348. (C) Correlation of HDACs and PARPs expression levels in pancreatic cancer tissues and normal tissues; n = 348. (D–M) GSEA for gene sets associated with the antigen presentation-, immune-, and activate T cell-related signaling pathways. (N) Heatmap showing RT–qPCR data for indicated genes in PANC-1 cells treated with Vorinostat, Olaparib, or their combination for 48 h; n ≥ 3.


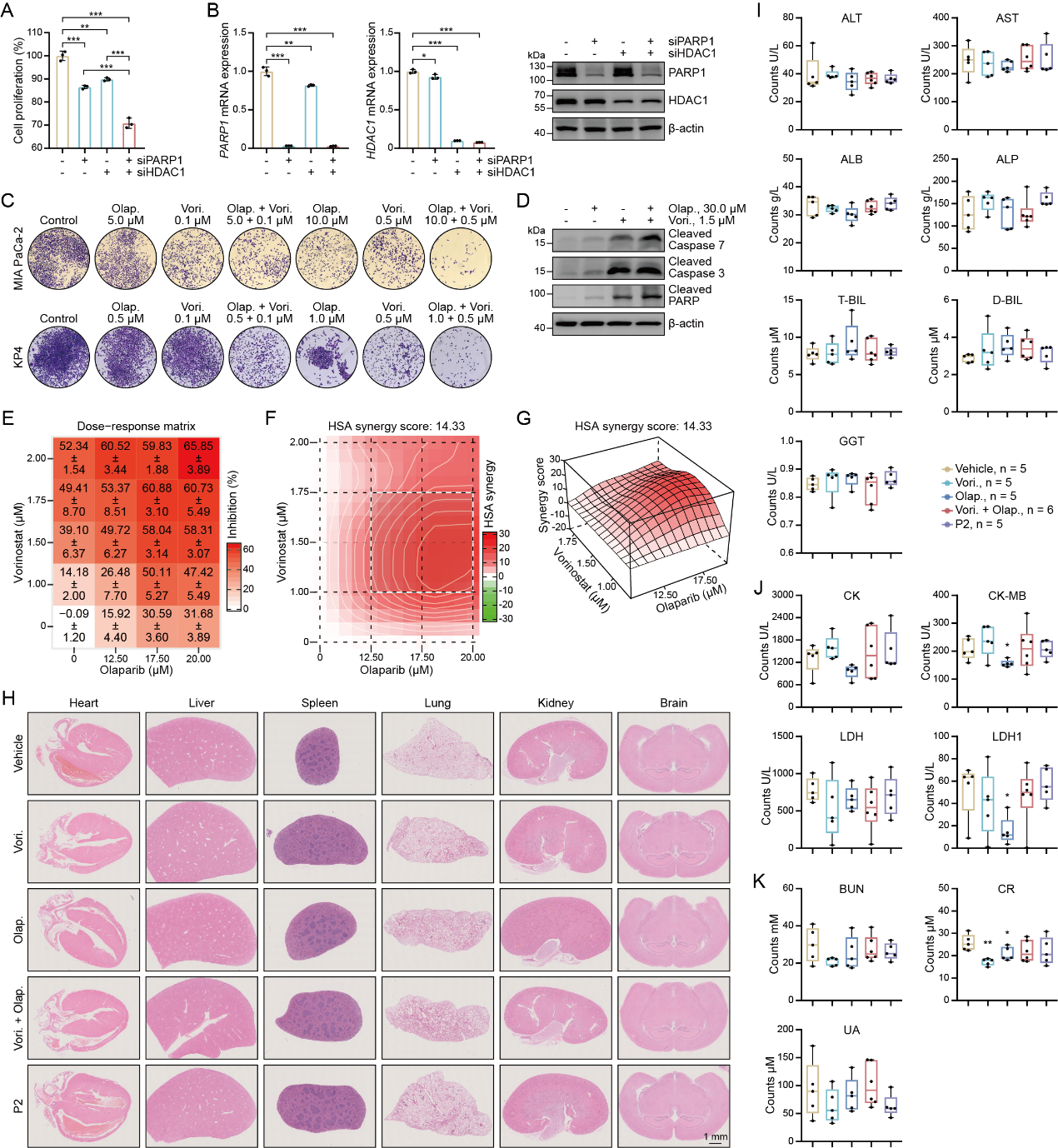


**Figure S4.** Combined HDAC and PARP inhibition synergistically elicited a potent antitumor effect. (A) The MIA PaCa-2 cells transfected with indicated siRNA for 72 h. Cell viability was assessed using an CCK8 assay; n ≥ 3. (B) RT–qPCR and immunoblot analyses of PARP1 and HDAC1 expression; n ≥ 3. (C) Pancreatic cancer cells treated with Vorinostat, Olaparib alone or in combination with indicated concentrations. Representative colony at magnification: ×50; n ≥ 3. (D) Apoptosis-related proteins expression levels were analyzed in MIA PaCa-2 cells treated with Vorinostat, Olaparib, or their combination for 72 h; n ≥ 3. (E–G) Combination study of the growth inhibitory effects of Vorinostat and Olaparib on MIA PaCa-2 cells. The above matrix was generated using SynergyFinder Plus and describes the percent inhibition for each treatment at each specific dose-response point, as well as the overall percent inhibition; n ≥ 3. (H) Mice were killed, histological examination of heart, liver, spleen, lungs, kidneys, and brain; n ≥ 5. (I–K) Hepatic, cardiac, and renal function parameters in mice treated with vehicle, Vorinostat, Olaparib, combined Vorinostat and Olaparib, and P2. ALT: alanine transaminase, AST: aspartate transaminase, ALB: albumin, ALP: alkaline phosphatase, T-BIL: total bilirubin, D-BIL: direct bilirubin, GGT: γ-glutamyltransferase, CK: creatine kinase, CK-MB: creatine kinase isoenzyme, LDH: lactate dehydrogenase, LDH1: lactate dehydrogenase isozyme 1, BUN: blood urea nitrogen, CR: creatinine, UA: uric acid; n ≥ 5. The data are expressed as mean ± SEMs; unpaired student’s *t*-test; **p* < 0.05; ***p* < 0.01; ****p* < 0.001.


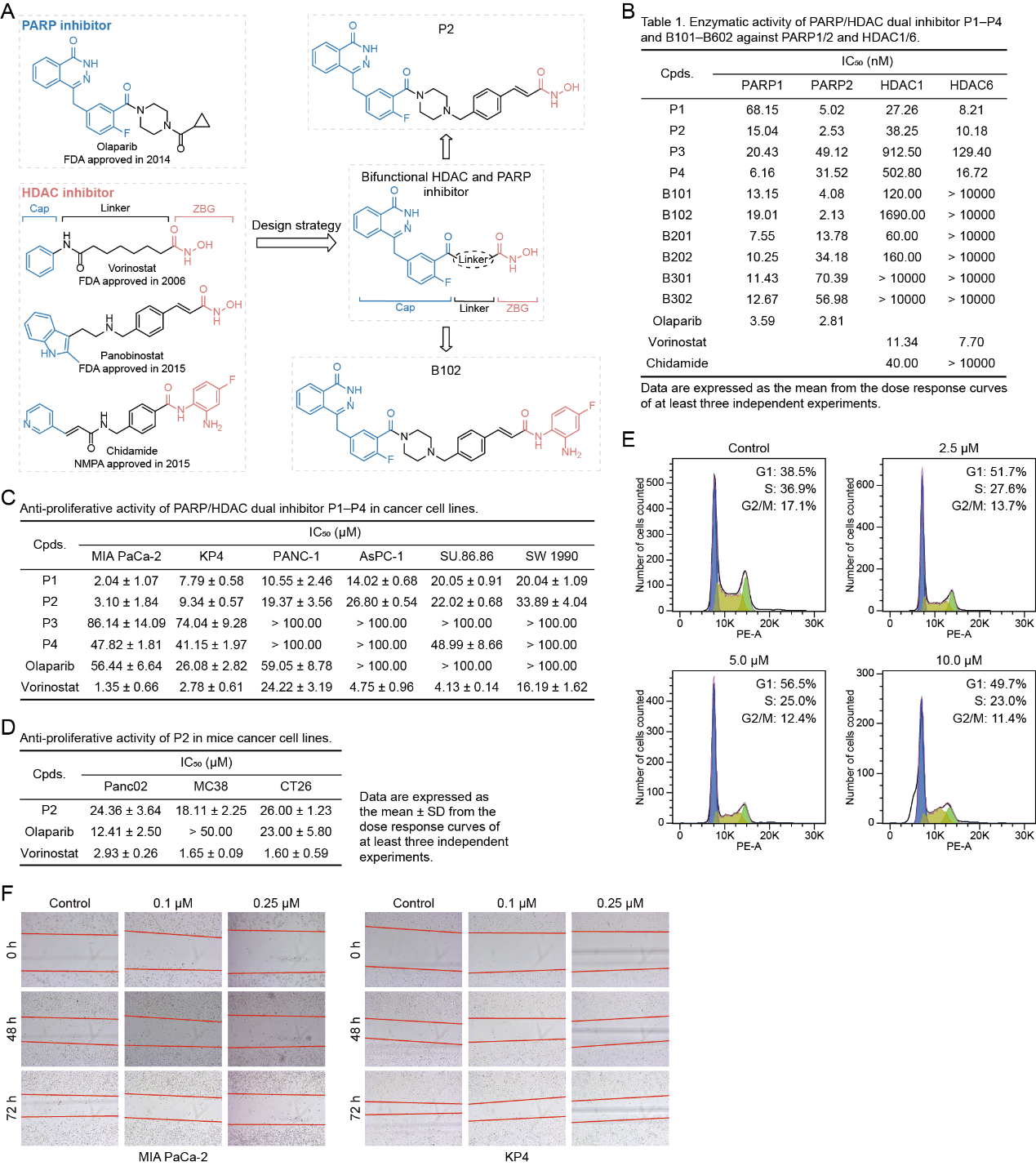


**Figure S5.** Bifunctional HDAC and PARP inhibitor exhibits potent antitumor effect. (A) Synthesis of designed bifunctional HDAC and PARP inhibitors. (B) Enzymatic activity of bifunctional HDAC and PARP inhibitor P1-P4 and B101-B602 against PARP1, PARP2, HDAC1, and HDAC6; n ≥ 3. (C–D) Anti-proliferative activity of P1-P4, Olaparib and Vorinostat in human cancer cell lines and mice cancer cell lines; n ≥ 3. (E) Flow cytometric analysis of cell cycle distribution in MIA PaCa-2 cells treated with P2 for 48 h; n ≥ 3. (F) Wound healing assay of P2 at different concentrations in MIA PaCa-2 and KP4 cells; n ≥ 3.


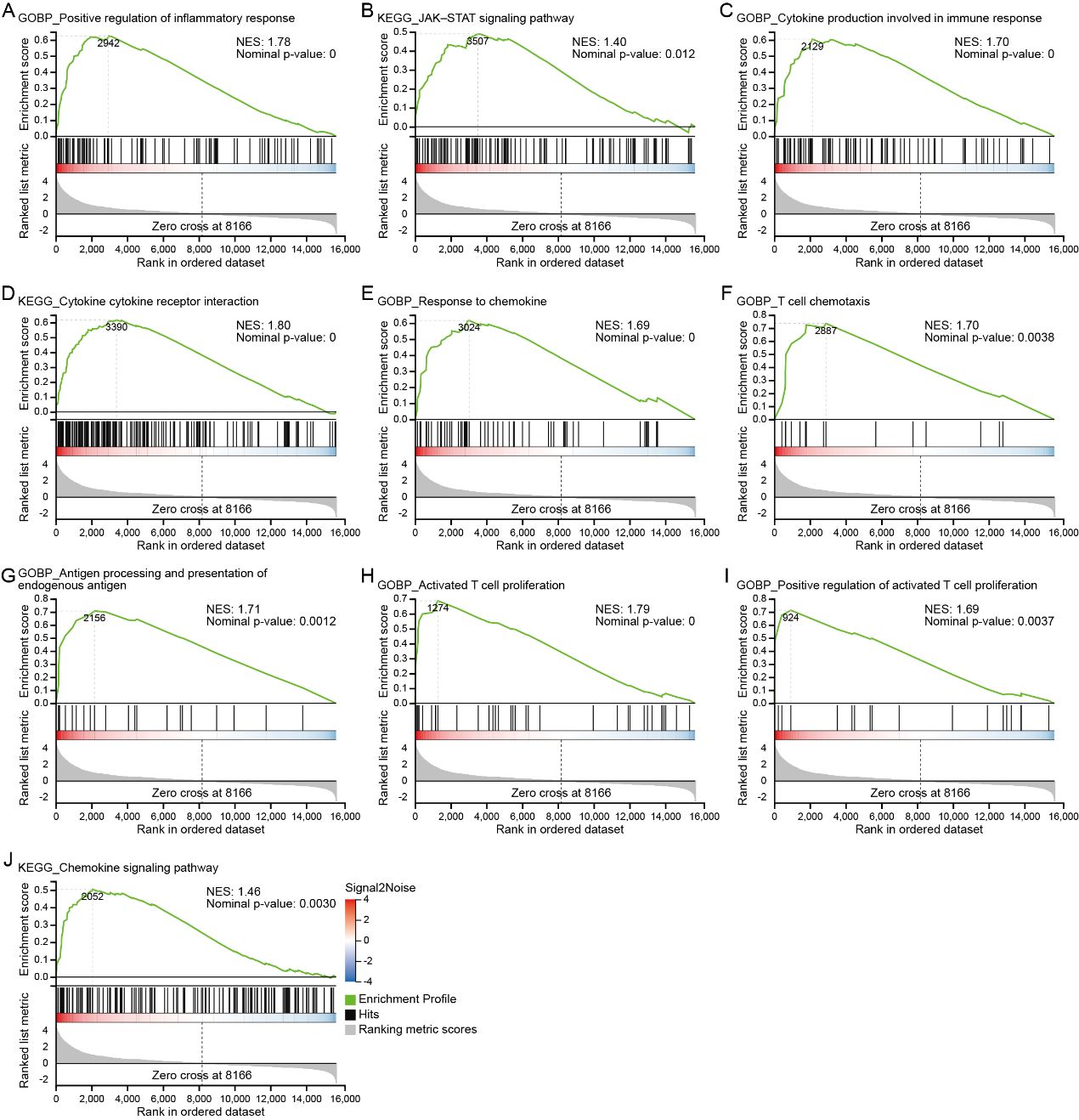


**Figure S6.** P2 can restore and activate STING signaling in *STING*-silenced tumor cells. (A–J) The MIA PaCa-2 cells treated with 5.0 μM P2 for 48h, GSEA for gene sets associated with the inflammatory response, JAK–STAT signaling pathway, innate immune signaling, antigen presentation-related signaling pathway and chemokine signaling pathway.


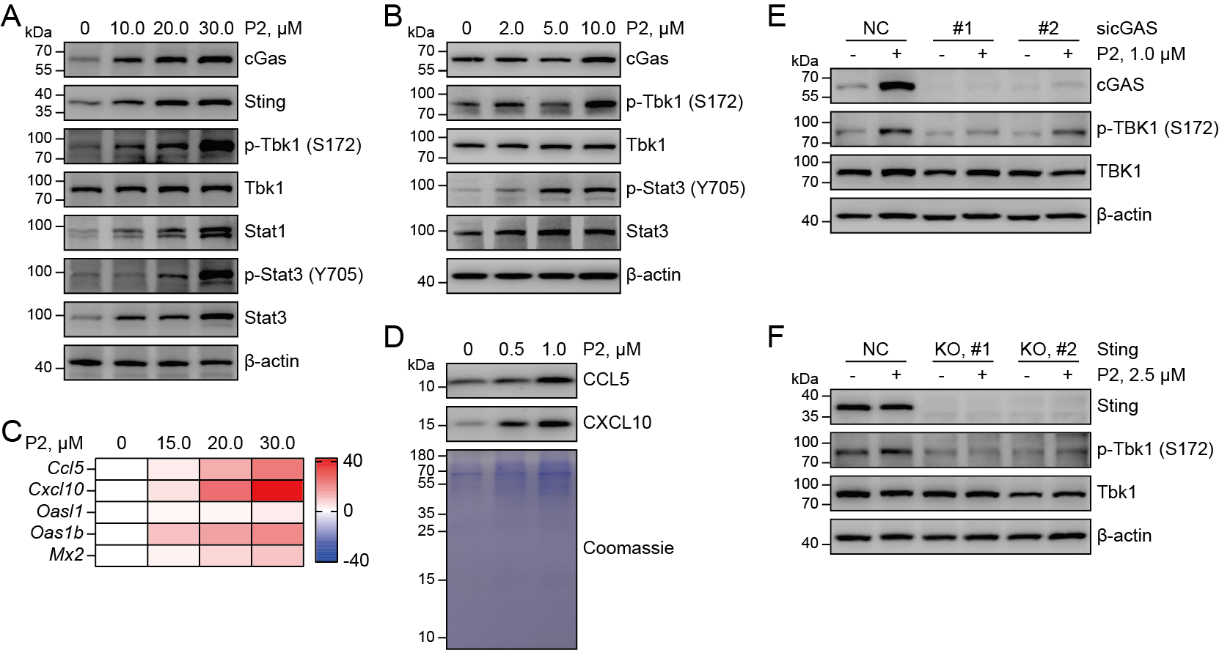


**Figure S7.** P2 can restore and activate STING signaling in *STING*-silenced tumor cells. (A–B) Expression of cGAS–STING signaling pathway-related proteins in Panc02 and MC38 cells treated with P2 for 48 h; n ≥ 3. (C) Heatmap showing RT–qPCR data for indicated genes in Panc02 cells treated with P2 for 48 h; n ≥ 3. (D) Analysis of secreted chemokines in P2-treated MIA PaCa-2 cells via immunoblotting; n ≥ 3. (E) p-TBK1 levels in *cGAS* knockdown MIA PaCa-2 cells treated with P2; n ≥ 3. (F) p-Tbk1 levels in *Sting* KO MC38 cells treated with P2; n ≥ 3.


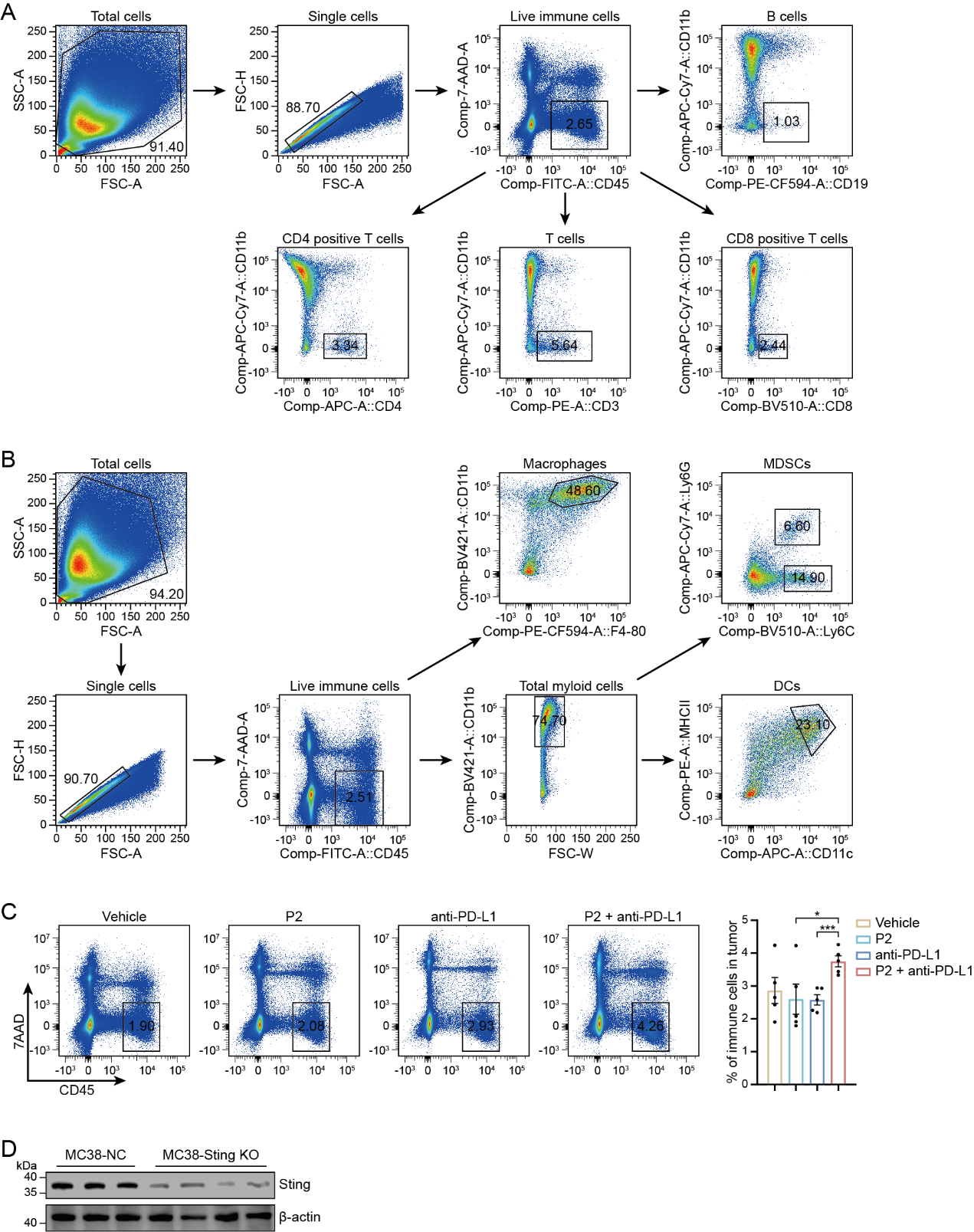


**Figure S8.** P2 enhances antitumor immunity by recruiting T cells and potentiates anti-PD-L1 efficacy in syngeneic models. (A–B) The gate rules of flow cytometry analysis in MC38 tumors. (C) The tumor infiltration of CD45^+^ T cells in MC38 tumors was detected by flow cytometry; n ≥ 4. (D) Tumor tissue isolated from MC38 wildtype and Sting KO mice, and western blot was performed to detect Sting levels; n ≥ 3. The data are expressed as mean ± SEMs; unpaired student’s *t*-test; **p* < 0.05; ***p* < 0.01; ****p* < 0.001.

# Supplementary Materials and Methods

## Chemicals

DNMTis: SGI-1027 (Selleck, S7276), Decitabine (Selleck, S1200); HDACis: Vorinostat (Selleck, S1047), Entinostat (Selleck, S1053), Panobinostat (Selleck, S1030), RGFP966 (Selleck, S7229); LMK-235 (MCE, HY-18998); MC1568 (MCE, HY-16914); Tubastatin A (MCE, HY-13271A); FT895 (MCE, HY-112285); OSS_128167 (MCE, HY-107454); BRD4i: (+)-JQ1 (Selleck, S7110); EZH2i: CPI-169 (Selleck, S7616); JMJD3i: GSK J4 (Selleck, S7070); DNMT/G9ai: CM272 (Selleck, S8812); JAK1/2i: Ruxolitinib (Selleck, S1378), JAK3i: Tofacitinib (Selleck, S2789). P2 was synthesized in our team. Inhibitors were dissolved in DMSO and stored at -20 °C.

## Cell lines and cell culture

MIA PaCa-2 (RRID: CVCL_0428), KP4 (RRID: CVCL_1338), PANC-1 (RRID: CVCL_0480), AsPC-1 (RRID: CVCL_0152), SU.86.86 (RRID: CVCL_3881) and SW1990 (RRID: CVCL_1723) cells were kindly provided by Cell Bank, Chinese Academy of Sciences (originally sourced from the American Type Culture Collection). MC38 and Panc02 cells were kindly provided by Zhongshan School of Medicine (Prof. Jun Chen, Sun Yat-sen University). MIA PaCa-2, PANC-1, MC-38, Panc02 and SW1990 cells were maintained in DMEM (Corning, 10-013-CV) with 10% FBS (Gibco, 10091-148) at 37 °C in a humidified atmosphere containing 5% CO_2_. KP4, AsPC-1 and SU.86.86 cells were maintained in RPMI 1640 (Corning, 10-040-CV) with 10% FBS at 37 °C in a humidified atmosphere containing 5% CO_2_. When the cells are at 80% density, they can be harvested for experiments. All cell lines were authenticated by Short Tandem Repeat (STR) profiling and routinely tested to ensure they were free of mycoplasma contamination.

## Cell viability assay

MTT kit (BBI, A600799-0250) and CCK8 kit (APExBIO, K1018) were performed on existing cancer cells to evaluate the antiproliferative activity of the inhibitors or cell viability after gene knocking down. Based on the cell growth rate, cells were inoculated at an appropriate density in a 96-well plate. After attaching overnight, cells were treated with specified drugs dissolved in DMSO at various concentrations for 72 h. Subsequently, MTT or CCK8 was added and incubated for 4 h at 37 °C and dissolved in 100 µL of DMSO per well. The absorbance at 490 nm or 450 nm was measured using the SpectraMax i3x enzyme marker. Three independent experiments were conducted, and IC_50_ values were calculated from the inhibition curves using Origin software (RRID: SCR_002815).

## Colony forming assay

MIA PaCa-2 and KP4 cells were spread in 6-well plates at a density of 200 cells per well and treated with the indicated compounds for 14 d, during which time the medium was changed and the corresponding concentrations of compounds were reintroduced every three days. Subsequently, the colonies formed were fixed at 37 °C with Bourne tissue fixing solution for 1 h, washed 3 times with PBS after fixation, dyed with crystal violet solution overnight, washed 3 times with ddH_2_O. The samples were dried and photographed using an inverted fluorescence microscope. Each sample takes three views.

## Wound healing assay

MIA PaCa-2 and KP4 Cells were s spread in 6-well plates at 70% convergence. After cells adhered, two perpendicular scratch wounds were created with 200 μL tip. Washing three times with PBS to remove the scraped cells, and FBS-free medium was added to slow down cell growth and proliferation, minimizing interference with migratory ability. Wound areas near the scratch lines were photographed at 0, 48, and 72 h.

## Transwell invasion assay

The invasion assay for MIA PaCa-2 and KP4 Cells utilized transwell chambers (24-well inserts, 8.0 μm, Corning, 3524). In the invasion assay, pancreatic cancer cells were seeded onto the upper chamber membrane in serum-free medium. The lower chamber was supplemented with complete medium containing 10% FBS to serve as a chemoattractant. After 48 hours of incubation, the noninvasive cells remaining on the upper chamber were removed. Subsequently, invasive cells on the submembrane surface were fixed using 10% (wt/vol) formalin and stained with crystal violet. Images were captured using a bright-field optical microscope equipped with a 10× objective lens.

## Western blot analysis

Pancreatic cancer cells and other cells were plated into cell culture dishes at suitable density to adhere overnight and received the corresponding concentrations of inhibitors treated for 24, 48, or 72 h. Following treatment, cells were lysed with ice-cold Nonidet P-40 buffer containing phosphatase and protease inhibitors (Roche, 04 906 837 001/04 693 132 001). Protein concentrations were quantified using the BCA Protein Assay Kit (Thermo, 23227), and equal amounts of protein were loaded onto polyacrylamide gels (EpiZyme Biotechnology, PG212) for analysis. After running, polyacrylamide gels transferred onto polyvinylidene fluoride membrane (Merck Millipore, IPVH00010), and blocked in 5% skimmed milk in TBST. Membranes were incubation with primary antibodies (1:1000) and secondary antibodies (1:2000) (Supplementary Table S1). Membranes were detected using an enhanced chemiluminescence reagent (Bio-Rad, 1705061).

## Immunofluorescence analysis

Pancreatic cancer cells with a density of 20% were seeded onto confocal petri dishes. After 12 h of cells attachment, the cells were treated with the corresponding inhibitors for 48 h. The cells were first washed with PBS, then fixed in pre-cooled methanol for 10 minutes at room temperature. After fixation, the cells were incubated in blocking buffer for 2 h at room temperature. Then, the cells were incubated with dsDNA antibody (1:200) and phospho-H2A.X antibody (1:200) diluted in an antibody dilution buffer (1% BSA, 0.3% Triton X-100, PBS) at 4 °C overnight. After the primary antibody incubation, cells are washed with PBS and incubated with Goat Anti-Rabbit IgG (1:200) or Goat Anti-Mouse IgG (1:50) diluted in the antibody dilution buffer for 1 h at room temperature. Since then, cells were washed again with PBS, mounted on dishes using ProLong Gold antifade reagent with DAPI (Yeasen, 36308ES20) and imaged using ultrahigh-resolution confocal microscopy (Zeiss, LSM880) at the designated magnification. The acquired images are analyzed using Zeiss software (RRID: SCR_021725) to quantify the fluorescence signals.

## Multiplex immunofluorescence staining

Multiplex immunofluorescence staining was performed on a commercially available human pancreatic cancer tissue microarray (Outdo Biotech), which included 69 tumor specimens. Tissue sections were first deparaffinized, rehydrated through graded ethanol, and subjected to heat-induced epitope retrieval using EDTA buffer (pH 9.0) in a pressure cooker for 10 minutes. After cooling to room temperature and washing with PBS, sections were blocked in antibody dilution buffer (1% BSA, 0.3% Triton X-100 in PBS) for 1 hour. Primary antibodies against STING (1:200) and CD45 (1:200) were diluted in antibody dilution buffer and incubated overnight at 4 °C in a humidified chamber. After three PBS washes, sections were incubated for 1 hour at room temperature with species-appropriate fluorophore-conjugated secondary antibodies. Nuclei were counterstained with DAPI. mages were acquired using the Pannoramic DESK digital slide scanner (3DHISTECH), and fluorescence signals were analyzed with ImageJ software (RRID: SCR_003070) to quantify target expression across tumor regions.

## Poly(I:C), Poly(dG:dC) and small-interfering RNA (siRNA) transfections

Poly(I:C) (APExBIO, B5551), Poly(dG:dC) (InvivoGen, tlrl-pgcn) and siRNA transfection were performed with RNAiMAX transfection reagent (Thermo, 13778150), followed the manufacturer’s instructions. The specific siRNA target sequences were synthesized by GenePharma (Supplementary Table S2).

## Lentivirus production and gene knockdown by short-hairpin RNA (shRNA)

The shRNA oligos, which contain sequences targeting specific genes as listed in Supplementary Table S3, were combined and inserted into either a pLKO.1-Puromycin+ (Puro) lentiviral vector through annealing and cloning processes. Lentivirus containing the pLKO.1 plasmid (RRID: Addgene_40069) was generated by co-transfecting 293T cells with helper plasmids pMD2.G (RRID: Addgene_12259), and psPAX2 (RRID: Addgene_12260). After a 48-h incubation period, the medium containing viruses were harvested and centrifuged at 1200 × g for 8 min. Collected viral fluids was dispensed into 1 mL tubes. This lentivirus can be used directly to infect cells with the addition of 8 mg/mL polybrene (Biotechnology, C0351) and the rest can be frozen at -80 °C for storage. To establish stable knockdown cell lines, cells of the wildtype were plated in 6 cm cell culture plate and then exposed to 1mL viral fluids with polybrene for transduction. After a 6 h incubation period, the incubation was extended for 24 h with the addition of an extra 2 mL of complete DMEM medium followed by puromycin selection for 2 weeks. Afterward, the expression of genes was analyzed and confirmed by RT–qPCR or western blot.

## RNA isolation and real-time quantitative polymerase chain reaction (RT–qPCR)

Cells were seeded into 6‐well plates and subjected to treatment with specified inhibitors. The Eastep Super Total RNA Extraction Kit (Promega, LS1040) was employed for total RNA extraction, followed by reverse transcription of the RNA into cDNA using HyperScript RT SuperMix (APExBIO, K1074). Quantitative real-time PCR was conducted utilizing Hieff qPCR SYBR Green Master Mix (Yeasen, 11202ES08) with the Applied Biosystems 7500 Fast Real Time System (ABI, 4351107). GAPDH was used as a control. The primer sequences were as Supplementary Table S4.

## RNA sequencing (RNA-seq) analysis

Total RNA from pancreatic cancer was extracted with Trizol (Thermo, 15596018). Under the same experimental conditions, collect three independent replicates of the samples. Following this, the samples were submitted to the Beijing Genomics Institute (BGI) for sequencing. Subsequently, the sequencing data was processed and analyzed utilizing the BGI multi-omics tool (https://biosys.bgi.com/#/report/login).

## Proteomics analysis

MIA PaCa-2 were plated into 6 cm dish, treated with 0.5 μM Vorinostat and 10.0 μM Olaparib in combination for 48 h. Cells were lysed using ice-cold Nonidet P-40 buffer, and 100 μg lysates were reduced and alkylated. Then, the lysates incubated with trypsin (Promega, V5111) overnight at 37 °C. The digested peptides were collected, StageTip C18 desalting and MS analysis (RRID: SCR_024531).

## ChIP analysis

The ChIP assay utilized the truChIP Chromatin Shearing Kit (Covaris, 520127) and SimpleChIP Plus Sonication Chromatin IP Kit (CST, 56383). Briefly, cells cultured in 15 cm dishes were washed twice with PBS, then crosslinked with 1% haploid formaldehyde for 10 min. The Glycine of Shearing Kit was added to quenches the cross-link reaction. Cells were washed with PBS and scraped into 15 mL tube. Pelleted cells were centrifuged to remove the supernatant and added to 1 mL of Lysis Buffer B in Shearing Kit to lyse plasma membrane for 5 min. Collect intact nuclei by centrifugation at 1700 × g for 5 min at 4 °C, and resuspend pellet with Wash Buffer C in Shearing Kit. Then, the nucleic fraction was isolated Shearing Buffer D3 in Water bath ultrasonic crusher (Covaris, M220). After sonication, the 100 µL chromatin was added 400 µL Chip buffer in IP Kit and 2% volume of chromatin sample is as Input stored at -20 °C. The rest of chromatin samples were incubated with Acetyl-Histone H3 (Lys9) antibody (1:50) or IgG in IP Kit with 30 µL ChIP-Grade Protein G beads in IP Kit incubate overnight at 4 °C. The protein-DNA complexes were then de-crosslinked by treatment with 5 M NaCl and Proteinase K for 2 h at 65 °C. Subsequently, DNA purification was carried out using spin columns following the manufacturer’s protocol. The enriched DNA was subsequently analyzed by ChIP–qPCR.

## Bone marrow-derived dendritic cells (BMDCs) isolation and activation assay

Bone marrow was isolated from the femurs and tibias of 8- to 12-week-old C57BL/6 mice. After treated with ACK lysis buffer (Sangon Biotech, B541001-0100) at 37 °C for 5 min to remove red blood cells, cells were incubated at 37 °C with 5% CO_2_ in 6-well plate at 2.5 × 10^6^ cells/well concentration with RPMI 1640 medium supplemented with 20 ng/mL of recombinant mouse granulocyte-macrophage colony-stimulating factor (GM-CSF, Peprotech, 83869-56-1), 5 ng/mL of recombinant mouse IL-4 (Peprotech, 214-14), 10% FBS and 1% penicillin-streptomycin. On day 3, the culture medium was changed and replenished to remove the non-adherent granulocytes. The suspension and loosely adherent immature DCs were harvested on day 5 for experiments.

For the coculture experiments, 2 × 10^5^ MC38 cells were seeded in 6-well plate for 12 h allowed to adhere, then immature BMDCs were added and cocultured with or without MC38 cells at the ratio of 2.5:1 in 2 mL culture media in the presence of DMSO or P2 for 48 h. The suspension and loosely adherent BMDCs were harvested and stained with APC anti-mouse CD11c (Biolegend, 117310), PE anti mouse-MHCⅡ (Biolegend, 107607) and Brilliant Violet 421 CD86 (Biolegend, 117310) for flow cytometry analysis (RRID: SCR_008520).

## Animal experiments

Six-week-old female C57BL/6J mice were obtained from Guangdong Medical Laboratory Animal Center. All experimental procedures were approved by the Institutional Animal Care and Use Committee of Shenzhen University Medical School and were conducted in accordance with established guidelines (Approval Number: AEWC-202300007). Mice were matched for age, sex and genetic background and were appropriately randomized. For MC38 WT cells and MC38 STING KO cells, 1 × 10^6^ cells in 50 µL PBS were injected. Panc02 cells, 2 × 10^6^ cells in 50 µL PBS were injected. Tumor volumes were calculated using the formula (length × width × height)/2 and monitored every 3 days. Mice were euthanized when tumors reached 1,000 mm^3^, and this was recorded as a death for survival analysis. P2, Vorinostat and Olaparib were dissolved in 7% DMSO combined with 43% PEG-300 in PBS. For immunotherapy, mice were given 100 µg anti-PD-L1 (BioXCell, 10F.9G2) via intraperitoneal when the tumor volume reached ~100 mm^3^. The experimental procedure is designed as shown in the corresponding schematic diagram. For the rechallenged assay, MC38 tumour-free mice were rechallenged with 5-fold (5 × 10^6^) MC38 cells on the opposite flank 2 months after first tumor inoculation, and age-matched naïve mice were used as controls.

## Hematoxylin and eosin (HE) staining

Tumor tissue and a series of organs including heart, liver, spleen, lungs, kidneys and brain are removed and fixed in Bourne tissue fixing solution. The fixed organizations were given to the Servicebio Technology Co., Ltd. to tissue fixation, embedding, sectioning, and dewaxing, dehydration and followed by staining with the HE.

## Inhibitor toxicity analysis

To assess the liver, kidney, and cardiotoxicity of compounds, blood was collected along in tubes and left at room temperature for 2 h to allow serum separation. The separated serum was given to the Servicebio Technology Co., Ltd. to detect alanine transaminase (ALT), aspartate transaminase (AST), albumin (ALB), alkaline phosphatase (ALP), total bilirubin (T-BIL), direct bilirubin (D-BIL), γ-glutamyltransferase (GGT), creatine kinase (CK), creatine kinase isoenzyme (CK-MB), lactate dehydrogenase (LDH), lactate dehydrogenase isozyme 1 (LDH1), blood urea nitrogen (BUN), creatinine (CR), and uric acid (UA) levels.

## Flow cytometry analysis

Apoptosis was assessed using Annexin Ⅴ-FITC/PI double staining, and cell cycle distribution was analyzed separately by PI staining and quantified as G1, S, and G2/M phases. To analyze the immune profile within tumor microenvironment, tumour tissues were harvested from mice after treatments and finely minced using surgical scissors. And then digested using HBSS buffer (Sangon Biotech) containing 10 mM HEPES, 10% FBS, 50 μg/mL DNase Ⅰ, 1 mg/mL dispase Ⅱ, 1.25 mg/mL collagenase D, 0.85 mg/mL collagenase V and 1% penicillin-streptomycin (Sigma-Aldrich), at 70 rpm speed, 37 °C for 45 min. The digested cells were washed and strained through 70 μm filter (Millipore) and centrifuged at 600 × g for 5 min at 4 °C. 5 × 10^6^ cells were counted, resuspended in FACS buffer (2% FBS, PBS) and incubated with mouse 0.2 μg anti-CD16/32 (BioXcell, 2.4G2) to block FcγR for 30 min on ice. Following by staining with 7-AAD viability dyes and selective monoclonal antibodies of cell surface markers (as listed in Supplementary Table S5) for 30 min on ice. Flow cytometry was conducted following a standard protocol using the BD FACSCanto II (BD Bioscience), and data analyzed with FlowJo 10.6 software (Tree Star) (RRID: SCR_008520). Gating strategy is shown in Supplementary Fig. S8.

## Bioinformatics analysis

Gene expression profiles and associated clinical data for patients were obtained from TCGA database. Spearman correlation analysis between HDACs and PARPs in tumor and adjacent non-tumor tissues was performed using R version 4.2.1 to explore the relationship between HDACs and PARPs. Kaplan–Meier progression-free survival curves for human tumors were generated using the Kaplan–Meier Plotter, stratified by STING1 expression levels with the optimal cutoff automatically selected [1].

## Statistical analysis

Statistical analysis was performed using student’s *t*-test and one-way ANOVA with GraphPad Prism software (RRID:SCR_002798). The data are expressed as mean ± standard error of the means (SEMs). Statistical significance was defined as ns, not significant; **p* < 0.05; ***p* < 0.01; ****p* < 0.001. Each independent experiment was repeated more than three times.

# Supplementary Tables

## Table S1. List of antibodies used for western blot and immunofluorescence.

| Antibodies | Source | Identifier | RRID |
| --- | --- | --- | --- |
| cGAS | CST | Cat# 15102S | AB_2732795 |
| STING (D2P2F) | CST | Cat# 13647S | AB_2732796 |
| Phospho-STING | CST | Cat# 50907S | AB_2827656 |
| RIG-I | CST | Cat# 4200S | AB_2175706 |
| MDA-5 | CST | Cat# 5321S | AB_10694490 |
| MAVS | CST | Cat# 83000S | AB_2927715 |
| TBK1 | CST | Cat# 3504S | AB_2255663 |
| Phospho-TBK1 | CST | Cat# 5483S | AB_10693472 |
| IRF3 | CST | Cat# 11904S | AB_2722521 |
| Phospho-IRF3 | CST | Cat# 29047S | AB_2773013 |
| IRF7 | CST | Cat# 39659S | AB_2942011 |
| STAT1 | CST | Cat# 14994S | AB_2737027 |
| Phospho-STAT1 | CST | Cat# 9167S | AB_561284 |
| STAT3 | CST | Cat# 9139S | AB_331757 |
| Phospho-STAT3 | CST | Cat# 9145S | AB_2491009 |
| PARP1 | CST | Cat# 9532S | AB_659884 |
| HDAC1 | CST | Cat# 34589S | AB_2756821 |
| BRCA1 | CST | Cat# 9010T | AB_2228244 |
| RAD51 | Absin | Cat# abs100449 | AB_3694083 |
| Phospho-Histone H2A.X | CST | Cat# 9718T | AB_2118009 |
| Acetyl-Histone H3 (Lys9) | CST | Cat# 9649T | AB_823528 |
| Histone H3 | CST | Cat# 4499S | AB_10544537 |
| Cleaved Caspase-9 | CST | Cat# 20750S | [AB_2798848](http://antibodyregistry.org/AB_2798848) |
| Cleaved Caspase-7 | CST | Cat# 9491S | AB_2068144 |
| Cleaved Caspase-3 | CST | Cat# 9664S | AB_2068144 |
| Cleaved PARP | CST | Cat# 9541S | AB_331426 |
| CCL5 | R&D | Cat# AF-278-SP | AB_354440 |
| CXCL10 | Abcam | Cat# ab214668 | AB_3694087 |
| PD-L1 | CST | Cat# 13684S | AB_2687655 |
| β-actin | Beyotime Biotechnology | Cat# AA128 | AB_2861213 |
| dsDNA antibody | Sigma | Cat# MAB1293 | AB_94097 |
| HRP-labeled Goat Anti-Rabbit IgG(H+L) | Beyotime Biotechnology | Cat# A0208 | AB_2892644 |
| HRP-labeled Goat Anti-Mouse IgG(H+L) | Beyotime Biotechnology | Cat# A0216 | AB_2860575 |
| HRP-labeled Donkey Anti-Goat IgG(H+L) | Beyotime Biotechnology | Cat# A0181 | AB_3073542 |
| Cy3-conjugated Affinipure Goat Anti-Mouse IgG(H+L) | Proteintech | Cat# SA00009-1 | AB_2814746 |
| FITC-conjugated Affinipure Goat Anti-Mouse IgG(H+L) | Proteintech | Cat# SA00003-2 | AB_2890897 |

## Table S2. List of siRNA target sequences.

| Target gene | 5’ - 3’ |
| --- | --- |
| sicGAS, #1 | GGAAGAAAUUAACGACAUU |
| sicGAS, #2 | GAAGAAACAUGGCGGCUAU |
| siBRCA1, #1 | CAGCAGTTTATTACTCACTAA |
| siBRCA1, #2 | CAGGAAATGGCTGAACTAGAA |
| siPARP1, #1 | GAGUCAAGAGUGAAGGAAATT |
| siHDAC1, #1 | GCGACTGTTTGAGAACCTT |
| siSTING, #1 | CCTCATCAGTGGAATGGAA |
| siSTING, #2 | CTGGCATGGTCATATTACA |
| Negative control | UUCUCCGAACGUGUCACGUTT |

## Table S3. List of shRNA target sequences.

| Target gene | Genetic sequence 5’ - 3’ |
| --- | --- |
| shPARP1, #1, F | CCGGCGACCTGATCTGGAACATCAACTCGAGTTGATGTTCCAGATCAGGTCGTTTTTG |
| shPARP1, #1, R | AATTCAAAAACGACCTGATCTGGAACATCAACTCGAGTTGATGTTCCAGATCAGGTCG |
| shPARP1, #2, F | CCGGGCTTCACATATCAGCAGGTTACTCGAGTAACCTGCTGATATGTGAAGCTTTTTG |
| shPARP1, #2, R | AATTCAAAAAGCTTCACATATCAGCAGGTTACTCGAGTAACCTGCTGATATGTGAAGC |
| shHDAC1, #1, F | CCGGGCCGGTCATGTCCAAAGTAATCTCGAGATTACTTTGGACATGACCGGCTTTTTG |
| shHDAC1, #1, R | AATTCAAAAAGCCGGTCATGTCCAAAGTAATCTCGAGATTACTTTGGACATGACCGGC |
| shHDAC1, #2, F | CCGGCCGCAAGAACTCTTCCAACTTCTCGAGAAGTTGGAAGAGTTCTTGCGGTTTTTG |
| shHDAC1, #2, R | AATTCAAAAACCGCAAGAACTCTTCCAACTTCTCGAGAAGTTGGAAGAGTTCTTGCGG |
| shHDAC2, #1, F | CCGGGCCTATTATCTCAAAGGTGATCTCGAGATCACCTTTGAGATAATAGGCTTTTTG |
| shHDAC2, #1, R | AATTCAAAAAGCCTATTATCTCAAAGGTGATCTCGAGATCACCTTTGAGATAATAGGC |
| shHDAC3, #1, F | CCGGCCTTCCACAAATACGGAAATTCTCGAGAATTTCCGTATTTGTGGAAGGTTTTTG |
| shHDAC3, #1, R | AATTCAAAAACCTTCCACAAATACGGAAATTCTCGAGAATTTCCGTATTTGTGGAAGG |
| shHDAC3, #2, F | CCGGGCACCCAATGAGTTCTATGATCTCGAGATCATAGAACTCATTGGGTGCTTTTTG |
| shHDAC3, #2, R | AATTCAAAAAGCACCCAATGAGTTCTATGATCTCGAGATCATAGAACTCATTGGGTGC |
| shHDAC5, #1, F | CCGGGACTGTTATTAGCACCTTTAACTCGAGTTAAAGGTGCTAATAACAGTCTTTTTG |
| shHDAC5, #1, R | AATTCAAAAAGACTGTTATTAGCACCTTTAACTCGAGTTAAAGGTGCTAATAACAGTC |
| shHDAC6, #1, F | CCGGCATCCCATCCTGAATATCCTTCTCGAGAAGGATATTCAGGATGGGATGTTTTTG |
| shHDAC6, #1, R | AATTCAAAAACATCCCATCCTGAATATCCTTCTCGAGAAGGATATTCAGGATGGGATG |
| shHDAC6, #2, F | CCGGGCCTACGAGTTTAACCCAGAACTCGAGTTCTGGGTTAAACTCGTAGGCTTTTTG |
| shHDAC6, #2, R | AATTCAAAAAGCCTACGAGTTTAACCCAGAACTCGAGTTCTGGGTTAAACTCGTAGGC |
| ShHDAC7, #1, F | CCGGGCTGATCTATGACTCGGTCATCTCGAGATGACCGAGTCATAGATCAGCTTTTTG |
| ShHDAC7, #1, R | AATTCAAAAAGCTGATCTATGACTCGGTCATCTCGAGATGACCGAGTCATAGATCAGC |
| shHDAC8, #1, F | CCGGGCGTATTCTCTACGTGGATTTCTCGAGAAATCCACGTAGAGAATACGCTTTTTG |
| shHDAC8, #1, R | AATTCAAAAAGCGTATTCTCTACGTGGATTTCTCGAGAAATCCACGTAGAGAATACGC |
| shHDAC11, #1, F | CCGGCCCGACGTGGTGGTATACAATCTCGAGATTGTATACCACCACGTCGGGTTTTTG |
| shHDAC11, #1, R | AATTCAAAAACCCGACGTGGTGGTATACAATCTCGAGATTGTATACCACCACGTCGGG |
| shSTING, #1, F | CCGGGTTTACAGCAACAGCATCTATCTCGAGATAGATGCTGTTGCTGTAAACTTTTTG |
| shSTING, #1, R | AATTCAAAAAGTTTACAGCAACAGCATCTATCTCGAGATAGATGCTGTTGCTGTAAAC |
| shSTING, #2, F | CCGGGCATGGTCATATTACATCGGACTCGAGTCCGATGTAATATGACCATGCTTTTTG |
| shSTING, #2, R | AATTCAAAAAGCATGGTCATATTACATCGGACTCGAGTCCGATGTAATATGACCATGC |
| shcGAS, #1, F | CCGGGATGCTGTCAAAGTTTAGGAACTCGAGTTCCTAAACTTTGACAGCATCTTTTTG |
| shcGAS, #1, R | AATTCAAAAAGATGCTGTCAAAGTTTAGGAACTCGAGTTCCTAAACTTTGACAGCATC |
| shcGAS, #2, F | CCGGCGTGAAGATTTCTGCACCTAACTCGAGTTAGGTGCAGAAATCTTCACGTTTTTG |
| shcGAS, #2, R | AATTCAAAAACGTGAAGATTTCTGCACCTAACTCGAGTTAGGTGCAGAAATCTTCACG |

## Table S4. Primers for RT–qPCR.

| Target gene | Forward | Reverse |
| --- | --- | --- |
| GADPH | TGGCCTTCCGTGTTCCTAC | GAGTTGCTGTTGAAGTCGCA |
| HDAC1 | CTACTACGACGGGGATGTTGG | GAGTCATGCGGATTCGGTGAG |
| PARP1 | CGGAGTCTTCGGATAAGCTCT | TTTCCATCAAACATGGGCGAC |
| STING | GAAATTATTCCTGCAAGCCAAT TT | TCACCCTTCTTTTTCATGTAG |
| IFNA | AATGACAGAATTCATGAAAGCGT | GGAGGTTGTCAGAGCAGA |
| IFNB | AGCAACAGCAAGGCGAAAA | CTGGACCTGTGGGTTGTTGA |
| IFNL1 | GAAGACAGGAGAGCTGCAAC | GGTTCAAATCTCTGTCACCACA |
| IFNL2 | TCCAGTCACGGTCAGCA | CAGCCTCAGAGTGTTTCTTCT |
| CCL5 | GCTGCTTTGCCTACCTCTCC | TCGAGTGACAAACACGACTGC |
| CXCL9 | AAGACCTTAAACAATTTGCC CC | TGCTGAATCTGGGTTTAGACAT |
| CXCL10 | GATGACGGGCCAGTGAGAA | GCTCGCAGGGATGATTTCAA |
| CT45A1 | GCACCTGTGGGAGGAAACG | CCTGACTGCAGTAGGTCCTTG |
| SPANXB1 | TGTGAATCCAACGAGGCCAACG | CCACTAGTATGGTCGAGGACTC |
| HLA-A | TCAGATAGAAAAGGAGGGAGTTACA | ACAAGCTGTGAGGGACACAT |
| HLA-B | CCTGAGATGGGAGCCGTCTT | CTCCGATGACCACAACTGCT |
| HLA-C | GGACAAGAGCAGAGATACACG | CAAGGACAGCTAGGACAACC |
| TAP1 | CTCTGGAAACCCTGTGCGT | GACCTTCCACTAGACCATGA |
| TAP2 | GACCTTCCACTAGACCATGAGC | GGAGGATTAAGATTAGTACGATGGT |
| LMP2 | CTATCCAGCGTACTCCAAAG | GAAAGACCAGTCCTTGCTGA |
| LMP7 | GGTCCTACATTAGTGCCTTACGG | CGCAGATAGTACAGCCTGCATT |
| B2M | AGACCTTTGGGCTGCCTTAT | TAGCCTCCCTCACTCCAAGA |
| OASL | GCAGAAATTTCCAGGACCAC | CCCATCACGGTCACCATTG |
| OAS2 | GCTTCCGACAATCAACAGCCAAG | CTTGACGATTTTGTGCCGCTCG |
| ISG15 | GCCTCAGCTCTGACACC | CGAACTCATCTTTGCCAGTACA |
| IFI44 | ATGGCAGTGACAACTCGTTTG | TCCTGGTAACTCTCTTCTGCATA |
| MX1 | GCCTCAGCTCTGACACC | CGAACTCATCTTTGCCAGTACA |
| PD-L1 | TCACTTGGTAATTCTGGGAGC | CTTTGAGTTTGTATCTTGGATGCC |
| cGAS | CACGAAGCCAAGACCTCCG | GTCGCACTTCAGTCTGAGCA |
| IFNG | AGCAACAGCAAGGCGAAAA | CTGGACCTGTGGGTTGTTGA |
| BRCA1 | GGAAATGGCAACTTGCCTAG | CTGCGAGCAGTCTTCAGAAAG |

## Table S5. List of antibodies used for flow cytometry.

| Antibodies | Source | Identifier | RRID |
| --- | --- | --- | --- |
| 7-AAD Viability Staining Solution | BioLegend | Cat# 420403 | AB_3694088 |
| FITC anti-mouse CD45 | BioLegend | Cat# 103108 | AB_312973 |
| PE/Dazzle 594 anti-mouse F4/80 Antibody | BioLegend | Cat# 123145 | AB_2564132 |
| APC anti-mouse CD11c Antibody | BioLegend | Cat# 117309 | AB_313778 |
| Brilliant Violet 421 anti-mouse/human CD11b Antibody | BioLegend | Cat# 101251 | AB_2562904 |
| APC/Cyanine7 anti-mouse Ly-6G Antibody | BioLegend | Cat# 127623 | AB_10645331 |
| Brilliant Violet 510 anti-mouse Ly-6G/Ly-6C (Gr-1) Antibody | BioLegend | Cat# 108457 | AB_2650931 |
| PE anti-mouse CD3 Antibody | BioLegend | Cat# 100205 | AB_312662 |
| APC anti-mouse CD4 Antibody | BioLegend | Cat# 100515 | AB_312718 |
| Brilliant Violet 510 anti-mouse CD8a Antibody | BioLegend | Cat# 100752 | AB_2563057 |
| PE/Dazzle 594 anti-mouse CD19 Antibody | BioLegend | Cat# 115553 | AB_2564000 |
| APC/Cyanine7 anti-mouse/human CD11b Antibody | BioLegend | Cat# 101226 | AB_830642 |
| Brilliant Violet 421 anti-mouse CD279 (PD-1) Antibody | BioLegend | Cat# 135221 | AB_2562568 |
| InVivoMAb anti-mouse CD16/CD32 | BioXcell | Cat# BE0307 | AB_2736987 |

# References

1. Kovács S. A., Fekete J. T., and Győrffy B., Predictive biomarkers of immunotherapy response with pharmacological applications in solid tumors. *Acta Pharmacol Sin*. 2023;44(9):1879-1889.
